# Effects of chemical and hydrological stress on the wing morphology of a damselfly

**DOI:** 10.1093/ee/nvaf112

**Published:** 2025-11-06

**Authors:** Ken M Mauser, Samiksha Paudel, Olivia Sigmund, Martin H Entling, Jürgen Ott, Carsten A Brühl

**Affiliations:** iES Landau, Institute for Environmental Sciences, University of Kaiserslautern-Landau, Landau, Germany; iES Landau, Institute for Environmental Sciences, University of Kaiserslautern-Landau, Landau, Germany; iES Landau, Institute for Environmental Sciences, University of Kaiserslautern-Landau, Landau, Germany; iES Landau, Institute for Environmental Sciences, University of Kaiserslautern-Landau, Landau, Germany; L.U.P.O. GmbH, Trippstadt, Germany; iES Landau, Institute for Environmental Sciences, University of Kaiserslautern-Landau, Landau, Germany

**Keywords:** aquatic-terrestrial linkage, odonata, emergence, chemical pollution, drought

## Abstract

Dragonflies and damselflies are exposed to various anthropogenic stressors in the aquatic-terrestrial ecosystem, which can affect their development and fitness. The symmetry of their wings, shaped during the aquatic larval stage, can serve as an indicator of environmental stress during development. Recent advances in computer-vision now provide the opportunity to standardize and enhance the precision of 2D assessments of entire wings, including many structural parameters, enabling a more reliable comparison of the effects of multiple anthropogenic stressors. We investigated the effect of 3 anthropogenic stressors on the fluctuating wing asymmetry of the damselfly *Coenagrion puella*: (i) Exposure to the agricultural insecticide chlorantraniliprole in a climate chamber experiment, (ii) alteration of the aquatic community with the mosquito control agent Bti (*Bacillus thuringiensis israelensis*), and (iii) altered hydrological regimes, both of which were applied in a floodplain mesocosm experiment in a full 2-factorial design. We found changes in wing size and several asymmetry parameters in response to the insecticide and altered hydrological regimes, whereas Bti treatment increased the number of cells in front wings. Our results show that damselflies’ wing morphology and symmetry can be affected by anthropogenically induced stress in aquatic ecosystems. The intensity of stressor effects varied across treatments, with altered hydrology causing the strongest changes in wing size and asymmetry.

## Introduction

Dragonflies and damselflies link freshwater and terrestrial ecosystems. Due to their amphibiotic biphasic life cycle, disturbances during the aquatic larval stage can carry over to affect adult behavior, reproduction and lifespan in the terrestrial phase (Tüzün and Stoks 2018, Samways et al. 2025). During the aquatic larval stage, damselflies are exposed to several anthropogenic stressors. In an agricultural landscape, micropollutants (eg pesticides) reach streams and ponds by artificial drainage, runoff, and erosion (Imfeld et al. 2021). In addition, nutrients from fertilisers are washed in, changing the chemical composition of the water up to the point of eutrophication (Schoumans et al. 2014). In the context of climate change, water levels and the frequency and intensity of flooding can change with regional patterns (Blöschl et al. 2019), whereas still waters in floodplain areas depend on water input from flooding. Aquatic macroinvertebrates can initially experience a density increase when the water volume is reduced, but prolonged drought conditions then lead to a decline in the abundance of more sensitive species, presumably due to habitat degradation (Herbst et al. 2019). In addition, anthropogenic water regulation, such as dam operation, can intensify drought conditions during dry periods (Khelifa et al. 2021). Floodplain water bodies show a strong and highly dynamic relationship with the river, for example through exchanging chemically different water depending on the river water regime (Gmitrowicz-Iwan et al. 2020). Consequently, climate change may alter the input of micropollutants into floodplain water bodies by modifying the frequency and extend of river water exchange with these systems. Another anthropogenically introduced stressor is Bti (*Bacillus thuringiensis israelensis*) to control mosquitoes, which can also impact the aquatic-terrestrial ecosystem (eg through the reduction of chironomids; Brühl et al. 2020). Although no direct effect of Bti on damselfly larvae is known, it was shown to alter the benthic macroinvertebrate community composition driven by a reduction of the number of chironomid, libellulid, and coenagrionid larvae (Gerstle et al. 2023).

As adults, damselflies prey upon flying insects and thus rely heavily on the functioning of their wings. Wing size and shape are environmentally mediated traits that directly influence flight performance, thereby determining a species’ capacity for foraging, predator avoidance, and dispersal (Rocha-Ortega et al. 2022). The fluctuating asymmetry (FA) of wings is the deviation from the perfect bilateral symmetry of wings (eg size and shape difference between left and right wings) and is used as a proxy of development stability (Benítez et al. 2020). Stress has been associated with increased fluctuating asymmetry of insect wings, including honey bees (Friedli et al. 2020), bumblebees (Hierlmeier et al. 2022) as well as dragonflies and damselflies (Stoks 2001). Environmental stress during development can disrupt gene regulatory networks, morphogen gradients, and cellular processes such as proliferation or apoptosis, thereby reducing developmental buffering and leading to increased FA (Takahashi et al. 2010, Juarez-Carreño et al. 2018, Blanco-Obregon et al. 2022). Since wing development in damselflies takes place in the aquatic larval stage, asymmetrical wings in response to aquatic stress could alter the role of damselflies in the terrestrial system. Although FA has been used as a proxy for environmental stress in damselflies, its suitability as a biomonitoring tool is still controversial due to behavioral influences and complex developmental dynamics (Hardersen 2000a, Ryazanova and Polygalov 2013). Moreover, short-term disturbances such as parasitism during sensitive developmental windows may also increase FA independently of environmental quality, as shown by a significant correlation between mite load and wing asymmetry in *Coenagrion puella* (Bonn et al. 1996). In addition, a study examining FA of another coenagrionid species (*Coenagrion scitulum*) in relation to fitness traits such as mating success, as well as genetic parameters like heterozygosity, have yielded in no significant associations (Carchini et al. 2001). However, FA can still be considered sensitive to environmental stress in insects, especially when the stressor’s biological relevance is confirmed through negative effects on fitness traits and when confounding environmental variation is minimized (Beasley et al. 2013). These methodological challenges and the complexity of stressor interactions highlight the need for more comprehensive and standardized approaches across experimental contexts differing in environmental complexity and control. Addressing such questions requires both controlled laboratory settings to disentangle direct physiological effects and field-based approaches that capture broader, community-mediated processes.

In studies of FA, traditional approaches have typically focused on straightforward morphological traits, such as wing length, wing area, and overall shape asymmetry, usually derived from linear measurements or landmark-based methods (Graham et al. 2010). Advances in computer vision now offer the potential for a more precise and reliable 2D-assessment of the whole wing in combination with analyzing a large sample size. Modern algorithms can automatically detect boundaries, identify junctions, and extract vein patterns (Eshghi et al. 2024) thereby extending FA analyses to asymmetries in cell count, cell shape, and vein-junction positions. These features build a complex network that provides support and rigidity to the wing membrane (Rajabi et al. 2016). Wing venation can considerably affect both the dynamic deformation of insect wings during flight (Rajabi et al. 2016) and their susceptibility to mechanical damage and abrasive wear (Rudolf et al. 2019).

The scrambling damselfly *C. puella* is a common species in central Europe, mostly univoltine and present in natural standing waters as well as in urban and agricultural areas (Wildermuth and Martens 2019). The ability of this generalist species to inhabit different habitats (Muséum National d’Histoire Naturelle and Office Français de la Biodiversité 2025) makes it a potential indicator for numerous anthropogenic stressors in freshwater ecosystems. Its broad distribution, high local abundance, wide ecological tolerance, and well-documented taxonomy and life-history fulfil key criteria of effective bioindicators, such as cost-efficient sampling and a consistent response at the population level (Holt and Miller 2010).

We investigated the effect of 3 anthropogenic stressors on the fluctuating wing asymmetry of *C. puella* in a laboratory and field perspective by (i) exposing larvae to the insecticide chlorantraniliprole in a climate chamber experiment, by (ii) treating ponds with Bti, and (iii) altering their hydrological regime in a field experiment using artificial outdoor floodplain mesocosms (FPM). We hypothesized (i) that the insecticide chlorantraniliprole negatively affects wing size and FA due to its neurotoxic mode of action, which may impair larval development and thereby reduce larval weight gain as well as wing size and wing symmetry of the emerged imagines. For the mosquito larvicide Bti, we expected that (ii) it has a negative impact on the wing size and FA due to food web interference as it causes a decline in mosquito and chironomid larvae. We further hypothesized (iii) a negative impact on the wing size and FA due to strong water level fluctuations and their disturbance of the benthic communities and crowding effects during low water. Since damselfly wings have a complex structure, we also investigate whether any stress dependent changes in the symmetry of the wing are uniformly spread or spatially dependent. Based on sex-specific differences in larval activity, life-history traits, and development (Mikolajewski et al. 2005), we included potential differences in wing symmetry between male and female damselflies in our study.

## Materials and Methods

### Climate-Controlled Chamber Experiment: Insecticide Chlorantraniliprole Exposure

Chlorantraniliprole (CAS Number 500008-45-7) is an anthranilic diamide insecticide, introduced in 2007. It acts on insect ryanodine receptors and causes excessive release of intracellular Ca^2+^ stores, resulting in death by paralyzing muscles and inhibiting feeding (Lahm et al. 2007). Its degradation is considered slow with a 50% dissipation time (DT_50_) in the water phase of 23.5 d, water-sediment DT_50_ = 170 d and soil DT_50_ (field) = 204 d (Pesticide Properties Database [PPDB] 2024). Globally, chlorantraniliprole plays an important role as it is routinely used in rice fields (Wei et al. 2019, CIBRC 2024). In Germany, it is approved for the use in different kind of vegetables, pome fruit and grape (BVL Bundesamt für Verbraucherschutz und Lebensmittelsicherheit 2015) against potato beetles (*Leptinotarsa decemlineata*), leafroller moths (Tortricidae), snout moths (Pyralidae), and other caterpillars (Lepidoptera). It has an acute 96-h 50% lethal concentration (LC_50_) of 4 µg/L for *Chironomus dilutus* and chronic 28 d no-observed-effect concentration (NOEC) in static water of 2.5 µg/L for *Chironomus riparius* (PPDB 2024). For *Daphnia magna*, an acute 48 h half maximal effective concentration (EC_50_) of 11.6 µg/L and chronic 21 d NOEC of 4.47 µg/L was determined (PPDB 2024). Its predicted environmental concentrations in freshwaters vary between 0.04 and 9.12 μg/L (European Food Safety Authority 2013). In a large-scale study across 101 sites of small lowland streams in Germany, chlorantraniliprole was detected in 68% of event-driven samples and 38% of grab samples with a mean concentration of 0.0008 and 0.0007 μg/L, and max concentration of 0.0225 and 0.1031 μg/L, respectively (Liess et al. 2021). A recent study in the Upper Rhine Valley in Germany, measuring different available water sources at non-target sites (eg puddles and small streams) showed 4 detections in off-field puddles with concentrations between 0.014 μg/L and 0.002 μg/L (Mauser et al. 2025b). On the basis of available effect data and measured environmental concentration data, we opted for the following 4 concentrations to ensure non-lethal effects: 0 μg/L, 2.5 μg/L, 10 μg/L, and 40 μg/L (0, 0.0025, 0.01, and 0.04 mg/L). To prepare a stock solution, chlorantraniliprole was first dissolved in acetone before tap water was added. The amount of acetone present in the solution with the highest target concentration was also added to the control. This resulted in an acetone concentration <100 μl/L in all groups, in accordance with the Organization for Economic Co-operation and Development (OECD) Guideline 211 (OECD 2012) which describes the standard test for assessing the reproduction and survival of *Daphnia magna* under chronic exposure conditions.

Larvae of *C. puella* (*n* = 160) were collected on 13 April 2023 with a net (mesh size = 1 mm) from ponds in Trippstadt (Environmental Education Centre LIBELLULA, 49°21'30.5″N 7°44'36.0″E) within the Palatinate Forest in Southwest Germany. Only healthy larvae (based on their moving and lack of observable injuries) were taken which had not reached the final instar (F-0) yet. Larvae were immediately transferred to the lab in multiple 10 L buckets with previously aerated tap water (aeration time at least 24 h) and then split individually to 250 ml glass beakers with 100 ml of similar prepared medium and randomized IDs. Before weighing (± 0.01 mg), individuals were dried thoroughly with laboratory tissues (Carl Roth GmbH + Co. KG, Germany) to remove as much contact water as possible. To prevent exposing the larvae to an abrupt temperature increase, all beakers were first kept at constant room temperature (20 °C) for 24 h to allow acclimatization. All beakers with larvae were then transferred to a WK 19’/+15-35 climate chamber (Weiss Umwelttechnik GmbH, Germany). For the duration of the experiment, the parameters in the climate chamber were set as follows: temperature of 23 °C, humidity of 80% and a lighting time from 7 am to 11 pm, maintaining a day/night cycle of 16/8 h. The temperature of 23 °C was chosen based on previously reported optimal larval growth rates for *C. puella* (Suhling et al. 2015).

At the start of the exposure phase on 21 April 2023, the larvae were transferred to previously prepared 250 ml beakers with 100 ml of aerated tap water and the chlorantraniliprole concentrations of 0, 0.0025, 0.01, and 0.04 mg/l (40 beakers each). A plastic net stripe (length = 15 cm, width = 1.5 cm, mesh width = 0.2 cm) was added to the beakers as climbing assistance for emerging damselfly larvae. The beakers were covered with a piece of fly screen net (1 mm mesh width) and rubber band. Initially, the water exhibited a pH of 7.45, dissolved oxygen content of 10.33 mg/L and conductivity of 129 μS/cm at measured temperatures of 23.4 °C (Multi 340i, WTW, Germany). To compensate for evaporated water, the beakers were filled up to 100 ml with aerated tap water after 7 d. Every day of the exposure, a 100 μl water sample was taken for every concentration, stored at −20 °C and later analyzed (Roodt et al. 2023) via high-performance liquid chromatography coupled to tandem mass spectrometry (HPLC-MS/MS, Agilent 1260 Infinity II HPLC and Agilent 6495C MS, Agilent, United States). The 160 larvae were exposed until the 5 May 2023 for a total of 14 d. Four individuals emerged before reaching 14 d of exposure (7, 10, 10, and 12 d of exposure) but included in the analysis. After the exposure, the larvae were weighed again (±0.01 mg) (PBA224I-1x, VWR, United States) to calculate the wet weight gain. Until emergence, the larvae were fed daily with 2 ml Artemia suspension (prepared from ArtemiaVita eggs, Algova, Germany). The beakers were checked several times per day, and adults that had emerged on the plastic net strips and completed hardening were collected, placed in glass tubes, and frozen at −40 °C until further measurements. The imagines were weighed (±0.01 mg) (PBA224I-1x, VWR, United States) and species identity and sex were checked. Six adult individuals belonged to the closely related *Coenagrion pulchellum*, with very similar larval morphology, and were excluded from the analysis.

### Floodplain Mesocosm: Indirect Chemical and Direct Hydrological Stress

The floodplain mesocosms (FPM) are located at the Eußerthal Ecosystem Research Station (49°15′14′′N, 7°57′42′′E; RPTU Kaiserslautern-Landau) within the Palatinate Forest in Southwest Germany. The studied FPMs were 12 units each 176 m^2^ and rectangular in shape (23.5 × 7.5 m), constructed in 2017 along the stream Sulzbach, ensuring natural colonization of biota (Manfrin et al. 2023). Each unit has steep banks at 3 sides and a flat water–land floodplain riparian area at the inflow. All units are connected to the Sulzbach from where they can be supplied with water through a pipe to regulate water levels if needed. The FPMs progressively deepen toward the outflow section, following a bed slope of 1:20. They are inhabited by communities of merolimnic aquatic insect larvae (including Diptera, Ephemeroptera, Trichoptera, and Odonata) as well as various amphibians and terrestrial arthropods (Stehle et al. 2022). A detailed description of the FPM can be found in Stehle et al. (2022).

Since 2020, Bti was applied in 6 of the 12 FPM units (AP 2, 4, 6, 8, 10, 12) at maximum field rates (2.88 × 109 ITU/ha, Gerstle et al. 2023) annually in April, May, and June once a month using a VectoBac WDG solution (Valent BioSciences Corporation, Illinois, United States). To mimic a realistic Bti application, the FPM units were flooded 3 times by increasing the water level from 30 to 50 cm from mid-April to the end of May (only in the years 2020 and 2021, see Gerstle et al. 2023), before the implementation of the hydrological regime 2022. In 2023, the application rate was intensified, to increase hunger stress in the system, to 7 fortnightly applications starting from 14 April 2023 using the same field rates (Schöndorfer et al. 2025). Bti was only applied under windless conditions to avoid drift between ponds.

In 2022, 1 yr before the damselfly sampling, an altered hydrological regime was used in 6 of the 12 FPM units (“altered hydrology,” AP 3,4,7,8,11,12) as additional stressor to simulate a climate change scenario during the developmental phase of the larvae (see Bauspiess et al. 2025). Briefly, from February to April 2022 (hydrological winter), the standard water level of the FPMs was set at 40 cm for both the control and altered hydrology FPMs. The water level of the deeper part of the FPM, opposite the inflow area, was used as a guide for water level changes. When the water level was low (20 cm), the inflow area fell dry (only half of the FPM under water). When the water level was high (70 cm) the inflow area was under water. Although top layer freezing of the FPM units can occur at the study site during winter, no ice formation was observed during the hydrological manipulation periods. Throughout the hydrological winter, the control FPMs were flooded 3 times (once every 4 wk), while the altered hydrology FPMs experienced 11 floodings (weekly), with water levels rising to 70 cm for 4 to 5 d during each flooding event. From May to August 2022 (hydrological summer), the standard water level for the altered hydrology FPMs was adjusted to 20 cm, while it remained at 40 cm for the control FPMs. During the hydrological summer, the control FPMs maintained the same hydrological regime as in winter, with a standard water level of 40 cm and floodings every 4 wk (4 floodings in total). In contrast, the hydrological regime for the climate change scenario FPMs was modified: the standard water level was reduced to 20 cm, and the flooding frequency was changed from weekly to every 4 wk, also resulting in 4 floodings. For both control and altered hydrology FPMs, summer floodings involved a rise in water level to 70 cm for 4 to 5 d.

To catch all insects, one large emergence tent (Greenhouse 4.5 × 3.0 × 2.0 m, vidaXL, Netherlands) was placed in the water–land floodplain area of each FPM from 22 May 2023 to 11 August 2023. In total, we collected 79 adult *C. puella* at the beginning of the emergence season on 13, 14, and 15 June 2023 with an insect net (mesh size = 1 mm). We selected damselflies depending on availability in the units and approximation of equal shares between treatments and sexes. This resulted in 40 individuals from Bti-treated FPMs (20 male, 20 female) and 39 from untreated FPMs (20 male, 19 female). In case of the altered hydrology, it resulted in 25 individuals from the altered hydrology FPMs (8 male, 17 female) and 54 from the control FPMs (32 male, 24 female). We then transferred the individuals into glass tubes and stored them in a cooling bag during the time in the field to reduce their activity and minimize wing damage. Subsequently, we froze them at −40 °C until further measurements.

### Wing Photos

**Fig. 1.**
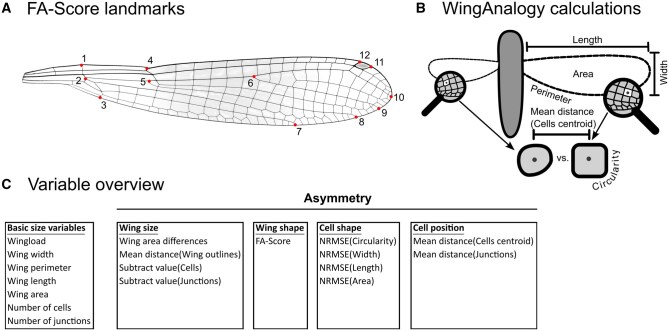
Calculation of geometric features. (A) Landmark placement to calculate the shape FA-Score. (B) WingAnalogy calculation of geometric features. The overall wing area, length, width and perimeter were compared between the left and right wing. In addition, several cells feature like the circularity were compared between the 2 sister cells between the left and right wing. The distance between cells centroid of sister cells added another cell position asymmetry parameter. (C) Overview of all wing variables, including basic size variables and asymmetry variables.

Photos of the imagines’ wings were taken with a stereomicroscope and camera (Stemi 508, Carl Zeiss AG, Oberkochen, Germany, objective magnification: 0.63×, internal zoom: 1.0×, camera adapter magnification: 0.5×) and attention was paid to achieve the best possible contrast between veins and white background by adjusting the light and exposure time as well as limiting the histogram values. Twelve landmarks (Lm) were set with the software IdentiFly (version 1.5, Tofilski 2023) on the hind antenodal cross vein (Lm 1 and 2), a branch of the anal vein (Lm 3), the nodus (Lm 4), the cubital vein (Lm 7), 3 branches of a medium vein (Lm 8, 9, 10), and 2 outer sides of the pterostigma (Lm 11, 12). Landmarks at positions 5 and 6 covered the wing interior along the second radius (Fig. 1A, Supplementary Fig. S1). Before setting landmarks on the wing photos, it was trained on 60 independent wing photos. Created landmark files were transferred to *R* for further analysis.

After removing cut cells at the wing base and particles from the image, we adjusted the contrast by using the *brightness/contrast* and *levels* tool in GIMP 2.10 (The GIMP Development Team 2023) and reduced the resolution to 1,000 × 563 pixels for stable computation and effective cell detection (Supplementary Fig. S2).

### Calculation of Geometric Features

We analyzed the wing images of front and hind wing pairs separately using WingAnalogy 4.0 (Eshghi et al. 2024), where vein coordinates were automatically aligned along the Y- and X-Axis based on the average outline distance. Each wing was divided into 2 sections (proximal section: Set 1, distal section: Set 2) at the branching of the second and third radius, with the cell column containing the branch origin included in Set 1 (Supplementary Fig. S3). We extracted overall geometric features of the wings (Fig. 1B), including the area, perimeter, length, and width as the mean between the left and right wing of a pair. Before calculating the asymmetry, we extracted the circularity of cells as well as the location of junctions, and the number of cells and junctions. Then, the normalized root mean square error (NRMSE) between the area, length, width, and circularity of corresponding cells between the left and right wing were calculated to describe the wing pair asymmetry of an individual. The asymmetry data output also included the mean distance between wing outlines, junctions, and cell centroids, as well as the difference in the number of cells and junctions (subtract value), wing area, and perimeter differences. A mean from the asymmetry comparison in both directions (left/right and right/left) was used for the further analysis. To discuss the cell shape asymmetry of each individual between the left and right wing, we further use the cell shape parameters (NRMSE of cell area, length, width, and circularity). To discuss the cell position asymmetry of each individual, we further use the cell position parameters (mean distances of junctions and cell centroids). For an overview of compared wing variables, see Fig. 1C. We excluded the regression and standard deviation values of the WingAnalogy output to reduce the number of variables and potential Type II error.

To calculate the overall wing shape asymmetry, the previously mentioned landmarks were used. First, a procrustes fit was performed in *R* for females and males separately with *gpagen* (geomorph package, version 4.0.7, Adams et al. 2024). FA and shape components were calculated with bilateral symmetry (Adams et al. 2024) and an individual wing pair wise FA score was calculated as distance between FA component and mean symmetric shape with *calc_fageo* (facefuns package, version 0.0.0.9, Holzleitner and DeBruine 2020). FA-score outliers were visually inspected and all were integrated into the analysis after the possibility of measurement errors has been excluded.

To calculate the wing load, we divided the adult wet weight by the mean wing area of either the front or hind wings, resulting in 2 values per individual. A normal distribution of the measured values ruled out anti-symmetry and a zero-mean ruled out directional asymmetry (Supplementary Fig. S4).

### Statistical Analyses

We used generalized linear models (stats package core, version 4.3.2, R Core Team 2023) for the response variables (see Supplementary Table S1) “Days until emergence,” “Wet weight,” “Wet weight gain,” “Landmark FA-score,” “Subtract value(Cells),” “Subtract value(Junctions),” “Wing area,” “Wings area differences,” “Wing length,” “Wing perimeter,” “Wing width,” “Number of cells,” “Number of junctions,” “NRMSE(Area),” “NRMSE(Length),” “NRMSE(Width)”, “NRMSE(Circularity),” “Mean distance(Cells centroid),” “Mean distance(Junctions)” and “Mean distance(Wing outlines)” and the explaining variable “concentration” (0 to 0.04 mg/l), and “sex” (f, m) to investigate the impact of the insecticide concentration. For the “Days until emergence,” “Number of cells,” “Number of junctions,” “Subtract value(Cells),” and “Subtract value(Junctions)” a Poisson distribution with log link was used. All other variables were fitted with a gaussian distribution with an identity link. Significance of the generalized linear model was tested with a *t*-test for gaussian family models and Wald-test for Poisson family models (base package core, version 4.3.2, R Core Team 2023). For the mesocosm analysis, we took the stressors treatment (non-bti/bti), hydrology (control/altered hydrology), their interaction and sex (f/m) as explaining variables with the same response variables (except “Days until emergence” and “Wet weight gain,” which were only available in the climate chamber experiment). Additionally, we applied likelihood-ratio χ^2^ tests (car package, version 3.1.2, Fox and Weisberg 2019) to assess the overall effect of categorical predictors and interactions in the generalized linear models of the mesocosm. The normal distribution of the residuals was visually checked for the models using Q–Q plots and the homoscedasticity and linearity were checked using residuals versus fitted plots, whereas no major irregularities were observed (stats package core, version 4.3.2, R Core Team 2023). Percentage changes were calculated as the relative difference between the model-predicted mean of each group and the baseline group mean. Standard errors were derived from the standard deviation of the model predictions within each treatment combination, divided by the square root of the number of predictions per combination, and scaled relative to the baseline mean.

The spatial wing analysis was conducted to localize which regions of the wing contributed most to overall asymmetry. For this purpose, individual cell data and coordinates were extracted from the WingAnalogy project file. The shape difference (eg cell circularity) or distance (eg between cell centroids) between the individual sister cells in the left and right wing and vice versa was calculated before the mean was taken. Sister cells were assigned automatically by WingAnalogy. All wings were superimposed using rotation over the upper edge of the wing and the center of gravity before they were further superimposed on the X and Y axes using translation, ensuring a uniform coordinate system for all wings. For a more uniform visualization, the lower and upper 5% was excluded for the cell circularity and width asymmetry. A matrix corresponding to the coordinate system with 300 x 300 cells was then created as a mean for all the wings of a group and interpolated with *interp* (akima package, version 0.6.3.4, Akima and Gebhardt 2022). The matrices were subtracted to calculate the differences between stressor and control. To remove noise in the data for better visualization, matrices were gaussian blurred with *isoblur* and a sigma setting of 10 (imager package, version 0.45.8, Barthelme 2024).

## Results

### Insecticide

The measured concentrations of chlorantraniliprole were 0.053, 0.014, 0.003, and 0 mg/L at the beginning of the exposure and stayed relatively constant over the course of the exposure (Supplementary Fig. S5). Of the initial 160 individuals, a total of 125 successfully emerged (0 mg/L: 33, 0.0025 mg/L: 30, 0.01 mg/L: 28, 0.04 mg/L: 34) which resulted in 106 complete front wing pairs and 110 complete hind wing pairs after the exclusion of damaged wings due to injuries during the emergence. The larvae needed 28.2 d on average from the beginning of the experiment until emergence (0 mg/L: 28.3, 0.0025 mg/L: 28.0, 0.01 mg/L: 28.0, 0.04 mg/L: 28.5).

**Fig. 2.**
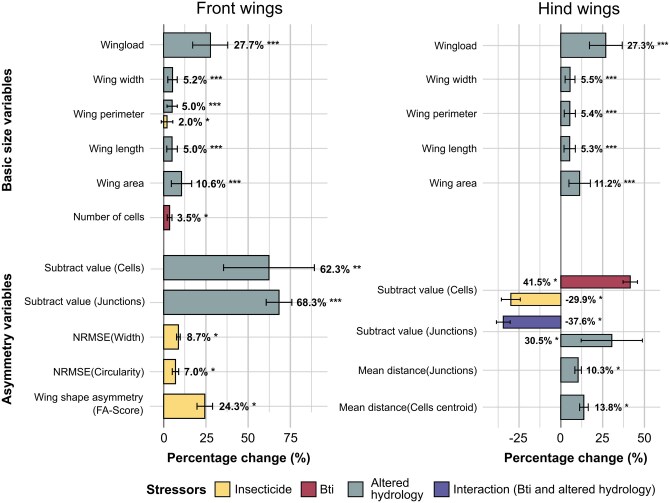
The 3 stressors insecticide (yellow), Bti (red), altered hydrology (gray) and interaction of Bti and altered hydrology (purple), and their significant effect sizes are shown as predicted percentage changes relative to the respective control group. Respective percentage changes are averaged over the levels of the other factors, thus also representing both sexes. Error bars indicate standard errors based on variation in model predictions. Interaction values represent the effect of Bti compared to the control group, calculated under altered hydrology. Values refer to different response variables of the wing size (load, width, perimeter, length, area, and number of cells) and wing asymmetry (Cell shape asymmetry: NRMSE(Width), NRMSE(Circularity); cell position asymmetry: Mean distance of cells centroids and junctions, wing shape asymmetry: FA-score; count asymmetry: Subtract values). The predicted effect of the insecticide applies to a concentration of 0.04 mg/L relative to the control (0 mg/L). To test for effects of the insecticide, a generalized linear model was constructed for each response variable, with insecticide concentration and sex as explanatory variables. To test for effects of Bti and the altered hydrology, a likelihood-ratio χ^2^ test was conducted for generalized linear models of each response variable, with Bti treatment, altered hydrology, their interaction and sex as explanatory variables. All model-based percentage changes, including nonsignificant ones, are shown in Supplementary Fig. S7.

Figure 2 provides an overview of the predicted effect sizes expressed as averaged percentage changes relative to the control group, offering a comparative perspective across all significant variables and both sexes. No differences between insecticide concentrations were detected for the days until emergence (z = 0.90, *P* = 0.369) or wet weight gain during the exposure (*t*_1,122_ = 0.06, *P* = 0.949). Male individuals had overall less wet weight gain during the exposure, smaller wings, and lower wing load in addition to different wing asymmetry compared to females (see Supplementary Table S2). At 0.04 mg/L, front wing perimeter significantly increased by 0.77 mm (estimate, *t*_1,103_ = 2.47, *P* = 0.015), and cell shape asymmetry significantly increased with the front wing NRMSE(Width) increasing by 0.028 (*t*_1,103_ = 2.08, *P* = 0.040) and NRMSE (Circularity) by 0.024 (*t*_1,103_ = 2.41, *P* = 0.018). The mean predicted increase in significant cell shape asymmetry variables for both sexes ranged from 7.0% to 8.7% (Fig. 2). In addition, the subtract value of hind wing cells significantly decreased by 0.70 (z = −2.40, *P* = 0.016) at the highest concentration. Moreover, the wing shape asymmetry was higher at 0.04 mg/L with a significant increase in the FA score in front wings by 0.003 (*t*_1,103_ = 2.49, *P* = 0.014) resulting in a predicted increase of 24.3% (Fig. 2). Nonsignificant predicted percentage changes can be seen in Supplementary Fig. S7.

### Bti and Altered Hydrology

After the exclusion of damaged wings, there were 26 front and 24 hind wing pairs of only Bti treated FPMs, 11 front and 10 hind wing pairs of only altered hydrology FPMs, 14 front and 13 hind wing pairs of the combined Bti and altered hydrology FPMs, and 52 front and 56 hind wing pairs of the control FPMs, which resulted in a total of 103 front and 103 hind wing pairs.

Mean predicted percentage changes of significant variables relative to the control groups for both sexes can be seen in Fig. 2. Similar to the insecticide, significant differences in several wing parameters were observed for Bti and altered hydrology. In Bti treatments, the number of cells in front wings significantly increased by 3.88 cells (estimate, χ^2^ = 4.75, *P* = 0.029) in addition to the subtract value of cells in hind wings (χ^2^ = 6.49, *P* = 0.011), resulting in a predicted increase of 3.5% and 41.5%, respectively (Fig. 2). There was also a trend of 1.17 mg heavier individuals (χ^2^ = 2.98, *P* = 0.084) and a higher subtract value of hind wing junctions (χ^2^ = 3.50, *P* = 0.061) in Bti-treated FPMs. Under altered hydrology, the body weight significantly increased by 3.36 mg (χ^2^ = 12.04, *P* < 0.001), front wing load by 0.10 mg/mm^2^ (χ^2^ = 22.53, *P* < 0.001), wing area by 5.33 mm^2^ (χ^2^ = 20.42, *P* < 0.001), wing length by 1.00 mm (χ^2^ = 19.11, *P* < 0.001), wing perimeter by 2.15 mm (χ^2^ = 20.83, *P* < 0.001), and wing width by 0.13 mm (χ^2^ = 18.32, *P* < 0.001). Predicted percentage changes due to altered hydrology for significant size variables ranged from 5% to 27.7% (Fig. 2). Similar significant effects of the altered hydrology on the wing size parameters were observed for the hind wings (Fig. 2, Supplementary Table S3). In addition, under altered hydrology, the wing asymmetry increased with significantly higher subtract value of cells in front wings (χ^2^ = 31.25, *P* = 0.001), subtract value of junctions in front wings (χ^2^ = 10.89, *P* < 0.001), subtract value of junctions in hind wings (χ^2^ = 6.36, *P* = 0.012) as well as the mean distance of cell centroids in hind wings by 21.28 μm (χ^2^ = 4.64, *P* = 0.031), and mean distance of junctions in hind wings by 16.37 μm (χ^2^ = 3.94, *P* = 0.047). Predicted percentage changes due to altered hydrology for significant cell position asymmetry variables ranged from 10.3% to 13.8% (Fig. 2). Nonsignificant predicted percentage changes can be seen in Supplementary Fig. S7.

There only was one significant interaction between Bti treatment and the altered hydrology for the subtract value of hind wing junctions (χ^2^ = 5.36, *P* = 0.021, Fig. 2). Post-hoc pairwise comparisons (Tukey adjustment) showed that individuals from the group with neither Bti nor altered hydrology differed significantly from all other groups. However, under Bti treatment, the positive effect of the altered hydrology was significantly lower (Bti and altered hydrology vs. non-Bti and altered hydrology, z = 2.969, *P* = 0.016). Male individuals showed smaller wings and less body weight and wing load in addition to a higher subtract value of front wing cells (see Supplementary Table S3).

### Correlation and Localization of Structural Changes across the Wing

In both front and hind wings, significant positive correlations were observed between all cell shape (eg NRMSE [Circularity]) and all cell position asymmetry variables (eg Mean distance (Cells centroid), Supplementary Fig. S6). In addition, wing size parameters (eg wing area) correlated significantly positively with the body weight and number of cells and junctions. The cell position asymmetry correlated positively with several wing size parameters, see Supplementary Fig. S6 for more details.

**Fig. 3.**
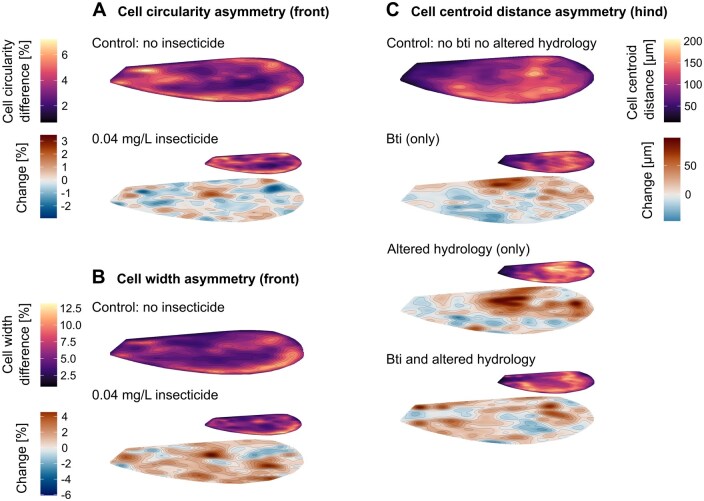
Spatial expression of wing cell asymmetry in response to environmental stressors. Each panel visualizes asymmetry between left and right wings in different traits and treatment scenarios, focusing on the spatial structure of trait responses. (A) Front wing cell circularity difference (%) (asymmetry) under insecticide exposure. (B) Front wing cell width difference (%) (asymmetry) under insecticide exposure. (C) Hind wing cell centroid distance (μm) (asymmetry) in response to Bti application and/or altered hydrology. In each panel, using a black-purple-yellow scale, the upper colored wing shows asymmetry values for the respective control group, while the small colored wing shows the treatment group. The red–blue colored wing illustrates the change in asymmetry (%) between control and treatment, where red areas define an increase in asymmetry under stress.

Under insecticide exposure, Bti treatment and altered hydrology various changes in wing morphology and asymmetry were observed, with its effects varying both individually and spatially, depending on the specific area or section of the wing (Fig. 3). The distribution of these morphological changes showed the possibility of shifts: asymmetry increased and concentrated in specific areas within the wing while it decreased in others due to the environmental alterations. When looking at the spatial distribution of the insecticide affected cell circularity (Fig. 3A), the cell circularity difference (asymmetry) between left and right wings in control wings (0 mg/L) was concentrated at the edges of the wing. Compared to the control, the cell circularity difference of the insecticide treated individuals changed in a spatially scattered manner, with no distinct pattern, except for cells at the wing base, central cells at the branching of the second and third radius and cells next to the pterostigma. The cell width difference (asymmetry) between left and right wings in control wings was mostly present at the outer edge of the wing (Fig. 3B) and below the pterostigma. The asymmetry of the insecticide-treated individuals was higher at the central cells after the branching of the second and third radius and at the same outer edge area compared to the control. When looking at the spatial distribution of the cell centroid distance asymmetry (Fig. 3C), the control asymmetry was positioned at the center of the distal wing part and the rear outer edge. The asymmetry of the Bti treated individuals was higher at the front outer edge and lower in the proximal half of the wing compared to the control. The altered hydrology showed a concentrated higher asymmetry at the branching of the second and third radius and at the same outer edge area where large cells form. The individuals under combined Bti and altered hydrology stress showed higher asymmetry at the center of the distal wing part compared to the control. Compared to individuals of Bti only, the similar patterned area in the lower proximal half of the wing was higher with an opposite effect.

## Discussion

We used a climate chamber and mesocosm experiment to study the effect of 3 different anthropogenic stressors on the wing morphology and asymmetry of the damselfly *C. puella*. The insecticide chlorantraniliprole caused an increase in asymmetry by means of the overall front wing shape (FA score) and cell shapes (NRMSE(width) and NRMSE(circularity)). The altered hydrology showed the strongest effects by increasing the wing size and load as well as the cell position asymmetry (mean distance of junctions and cells centroid) but not the overall wing shape. The mosquito control agent Bti increased the overall number of cells by 3.5%. The stressors affected individual traits and asymmetry to varying degrees, with spatially distinct effects across the wing. We conclude that anthropogenic stressors in aquatic environments influence damselfly wing morphology and asymmetry, suggesting a potential pathway of impacts into the terrestrial phase if these traits are functionally linked to terrestrial performance.

### Insecticide Effects

Supporting our first hypotheses, the insecticide caused an increase in asymmetry of the front wing shape (FA score) and cell shapes (NRMSE(width) and NRMSE(circularity)). Unexpectedly, the insecticide increased overall front wing perimeter and left other wing size parameters rather unaffected. The larvae were exposed for 2 wk in their late larval stages which represents a relatively small time window in the life of the damselfly which is usually around 1 yr in duration under field conditions, with larvae hatching between mid-July and late August, overwintering in instars 6 to 9, and emerging the following summer (Waringer and Humpesch 1984). Nevertheless, damselfly larvae were exposed during the final steps of wing development as an additional sensitive point of development at which stress can induce asymmetry: Larvae initiate critical pre-emergence processes such as wing sheath expansion and onset of melanization, indicating active wing tissue differentiation and structural maturation before emergence (Okude et al. 2021). This timing may amplify the effects of environmental stressors. The duration and timing of exposure are important. Prolonged exposure to a stressor can either cause an increase in asymmetry through sublethal, non-selective effects on development, or lead to lethal developmental selection for symmetry, which reduces levels of FA in the population (Beasley et al. 2013). The latter is especially the case for organisms with a stressful metamorphosis, which can erase the link between stress in the larval stage and adult asymmetry not by filtering out less fit individuals but by inducing an overall increase in asymmetry during metamorphosis itself (Campero et al. 2008). This pattern likely reflects the physiological challenges during emergence, where limited resources may be shifted toward processes of structural maturation, leaving reduced capacity to maintain developmental stability. As a result, metamorphosis acts as a levelling process that overrides prior stress signatures and leads to similar asymmetry levels across individuals regardless of their larval history. In our case, the insecticide exposure may have overlapped with a time window in which the development of the wings was already very advanced but the paralyzing mode of action may have complicated the final formation of cell/vein shape or overall wing shape by partially affecting the wing inflation process. Since in reality the larvae can be exposed to pesticides for longer periods of time, a possible effect on FA could be amplified under such conditions. Given the observed higher asymmetry of the front wings in *C. puella*, exposure to sublethal insecticide concentrations should be considered a factor that increases developmental stress and may directly affect the organism, either through morphological changes of important wing traits or through the higher energy demands needed to compensate for asymmetrical development. Pesticide exposure could have increased energy expenditure because of raised investment in detoxification and repair processes (Monteiro et al. 2019) and therefore shifted resources away from regulating developmental stability and maintaining bilateral symmetry. Maintaining a uniform cell size in all directions and maximizing area while minimizing material use is strongly determined by cell circularity (Rudolf et al. 2019). If maintaining equally high cell circularity—and thus material efficiency—across both wings is energetically demanding, in line with the theory of energetic costs of symmetry (Møller and Swaddle 2023), then deviations from symmetrical cell circularity could serve as indicators of larval developmental stress. Such stress might further trigger other behavioral responses associated with larval energy investment. An increase in asymmetry due to pesticide stress has been shown for coenagrionid larvae legs (Chang et al. 2007, Campero et al. 2008) but results for adult wings remain inconsistent (Hardersen et al. 1999, Hardersen 2000b, Campero et al. 2008). In our study, the insecticide exposure during the last larval stages led to a decrease of 0.70 subtract values of cells, with the effect being significant but confined to a small number of wing cell ­differences. Two individuals out of 29 control individuals with high subtract values of cells of 11 to 14 mainly drove this difference, probably explaining the observed effect. Individuals with high subtract values may have failed to emerge–similar to the negative effect on the subtract value observed under ­flooding conditions–which could suggest a selective mortality against highly asymmetric phenotypes due to the insecticide. However, this remains speculative, as no significant lethal effects were observed and the 6 mortalities at the highest concentration were comparable to those in lower concentrations and the control.

The increase in wing shape and cell shape asymmetry in our study was mainly driven by the highest insecticide concentration of 0.04 mg/L which is not in the range of environmental 1-time measurements in the same area (<0.014 μg/L, Mauser et al. 2025b). However, the environmental occurrence of this specific insecticide in combination with other complex pesticide mixtures over a longer time period could lead to sublethal effects on damselflies, that are not considered in pesticide authorization procedures. Nevertheless, they may alter key life-history traits, such as development, mobility, or reproduction success, which are essential for maintaining viable populations (Samways et al. 2025). Morphological alterations may have consequences for reproductive success, as recent work on *C. puella* demonstrated that wing morphology can be linked to short-term mating dynamics, although the direction and strength of this relationship may vary depending on local ecological conditions (Mauser et al. 2025a).

### Altered Hydrology Effects

Contrary to our hypotheses, the altered hydrology showed the strongest effects by increasing the wing size and wing load instead of decreasing it. However, in accordance with our hypotheses, cell position asymmetry (mean distance of junctions and cells centroid) and cell count asymmetry (subtract value of cells and junctions) was higher in altered hydrology FPMs. Lower water depth and a reduced water volume, as it was simulated in the climate change scenario, may have led to higher temperatures and wider temperature fluctuations. A higher water body temperature can result in decreased egg development and larvae with smaller body size but increased growth rate after hatching (Frances et al. 2017). This increased growth rate could then be limited by a trade-off with asymmetrical development (De Block et al. 2008), as we observed it for the higher mean distance of junctions and cells centroid as well as the higher subtract value of cells in the altered hydrology. *C. puella* populations can be partly semivoltine (ie individuals may take more than 1 yr to complete their development), as observed for more northern populations or populations with high larvae density (Wildermuth and Martens 2019). Since a larger wing size was recorded in the altered hydrology, a limitation of growth due to temperature stress seems only likely as a result of larvae remaining in the ponds for another year and then emerge delayed with an extended growth phase and resulting larger wings. This would imply that water temperatures exceeded a supportive level, as moderately warmer conditions have been shown to promote univoltine over semivoltine development in damselflies (Raczyński et al. 2022). However, this scenario appears unlikely in our case, since no significant differences in the abundance of *C. puella* were recorded in the previous year (Schleihauf, not published), which would be expected if a substantial portion of the population had delayed emergence. Further changes in the water due to lower water levels could have led to a change in the development of *C. puella*: As volume declines, major changes can occur in habitat quality like the increase in individual density, temperature, conductivity (salinity), turbidity, and major nutrients (eg nitrogen and phosphorus) as well as the decrease of dissolved oxygen (Lake 2011). Habitat change can lead to adverse effects on food resources by altering their availability, quality, and spatial distribution within the water body, which in turn affect the consumers and community structure (Herbst et al. 2019). If prey density increased at lower water levels, *C. puella* could have benefited in their early larval stages in the year before emergence, which could explain the larger wing size due to fostered development. In addition to prey availability, the survival of *C. puella* larvae also strongly depends on shelter conditions: they typically inhabit shallow shoreline areas or vegetation where the availability of cover is critical for reducing mortality under high larval densities (Wildermuth and Martens 2019). Thus, both prey concentration, cannibalism and microhabitat structure may have influenced larval development under the altered hydrological regime, albeit via different mechanisms. In the FPMs with high water level dynamics, we observed change of the vegetation over the course of the flooding year with the shallow ends of the pools becoming more overgrown (floodplain area), especially with horsetail (*Equisetum*). Such changes in vegetation structure may have influenced microhabitat conditions for *C. puella*, potentially relating to altered patterns of developmental stability. Predator presence (eg Aeshnidae) is a common factor, determining the activity level and food foraging of coenagrionids (Mikolajewski et al. 2005). Reduced density of the top predator *Aeshna cyanea* in the altered hydrology, as it was recorded in the year before sampling (Schleihauf, not published), may further supported the development of *C. puella* and its wing size. A trade-off between growth and developmental instability in terms of increased fluctuating asymmetry (De Block et al. 2008) may explain the increase in both wing size and cell position asymmetry beyond their positive correlation due to scaling effects alone. In addition to larger wings in the altered hydrology, the wing load increased, which can be directly connected to the flight speed and endurance (Gyulavári et al. 2017). As shown for the scrambling damselfly *C. puella*, flight endurance, rather than flight speed, is the main target of sexual selection (Gyulavári et al. 2014). Individuals under hydrological alteration showed larger wings but also higher wing load, which has contradictory implications for flight performance and mating success. While larger wings may improve aerodynamic efficiency and potentially support flight endurance, higher wing loading generally increases flight speed but may reduce endurance (Gyulavári et al. 2014). If endurance is a key factor for mating success, higher wing load may not necessarily be beneficial. However, it is unclear to what extent the observed increase in wing size under altered hydrology may influence this relationship. Alternatively, if flight speed plays a larger role, higher wing loading could provide an advantage, highlighting potential differences in selective pressures. While altered hydrological conditions may result in short-term physiological shifts, such as increased body mass, wing size, and wing load, it is difficult to assess whether these changes are beneficial or detrimental for *C. puella*, as long-term selective consequences for flight performance and reproductive success remain uncertain. Even if *C. puella* benefits as a generalist species that copes better with hydrological alterations, a community shift in dragon and damselflies with a decline of more specialized species must be considered, as generalists tend to be favored under environmental change while specialists face increased extinction risk (Cerini et al. 2020). As shown, hydrological alterations can influence the morphological development of damselflies, which may also be linked to other indirect habitat alterations and species composition in the water body. Due to the overwintering of *C. puella*, hydrological alterations during early larval stages in spring and early summer resulted in morphological responses that became apparent upon adult emergence in the following year. This time-lag highlights the importance of considering delayed effects when assessing the ecological impacts of environmental change. In the context of climate change, the consequences of altered hydrology can extend beyond the aquatic environment, affecting adult stages after emergence. These cross-ecosystem impacts should therefore be explicitly considered in conservation planning.

### Bti Effects

Contrary to our hypotheses, the mosquito control agent Bti significantly increased the mean number of cells per front wing by 3.5%, which is around 3 to 4 cells. Moreover, individuals emerging from Bti-treated FPMs tended to be heavier, with an average wet weight increase of 1.17 mg (∼5% averaged over sexes). In addition, Bti increased the asymmetry by increasing the subtract value of cells, which would support our hypotheses. However, other asymmetry parameters did not significantly respond to the Bti treatment. Bti is known to not only impact the target mosquitoes but also to reduce the amount of non-biting midges (Chironomidae) (benthic kicknet sampling: Gerstle et al. 2023, emergence traps: Kolbenschlag et al. 2023) and overall Odonata abundance and species richness (Jakob and Poulin 2016). A reduction of Libellulidae by 54% and Coenagrionidae by 27% due to Bti was observed at the FPM study site by collecting exuviae 2 yr before our sampling (Gerstle et al. 2023). However, no significant effect of Bti on the abundance of *C. puella* was found in the year before sampling (emergence traps: Schleihauf, not published) and in the abundance of chironomids in the year of sampling and the year before (emergence traps: Schöndorfer et al. 2025). Aquatic odonate larvae can follow 2 different strategies: “sit-and-wait” species prioritize avoiding predators, resulting in slower growth, and actively foraging species that accept higher predation risks in exchange for faster development (Harvey and White 1990). The larvae of *C. puella* exhibit pronounced behavioral plasticity: depending on predation risk and the availability of perches, they reduce their active foraging to minimize the likelihood of detection and can therefore react flexibly to environmental conditions (Convey 1988). This underscores, that the impact of Bti on the complex aquatic food web of odonates and other participating predatory species (eg Nepomorpha, Hydrophilidae, and Hydrachnidiae) may depend on multiple factors and effects could occur immediately or with a delay. If Bti reduced chironomid abundance during the larval stages of sampled damselflies but it was not detectable via emergence traps, there could have been an increase in cannibalism or intraguild predation (see Gerstle et al. 2024). Smaller damselflies in early larval stages experience more cannibalism (Anholt 1994), which would have favored larger individuals by filtering out smaller ones and by reducing resource competition. This would be in line with our findings of an increased number of cells and body weight. More cells may require a higher number of supporting veins, thereby increasing material costs. Plastic responses due to resource availability have been observed in *Drosophila melanogaster*, where poor nutrition led to reduced wing size, which resulted from decreases in both cell size and number (Vijendravarma et al. 2011). From another perspective, Bti could also have had an effect independent of the direct abundance of chironomids in the system, such as changing the community composition of chironomids and thus the nutrient availability through prey for *C. puella*. In combination with the altered hydrology, however, further Bti effects may have been partly blurred, since both treatments may have influenced other species that are indirectly related to the development of *C. puella* or by simply washing out the Bti and other prey or competing organisms by draining the water. The observed significant interaction of the treatment and altered hydrology, showed a slight suppression of the effect of the scenario on the subtract value of junctions in combination with Bti. This interaction was likely driven by 2 control individuals with high subtract values of 11 to 14, which may have led the interaction to reach statistical significance.

### Assessing Wing-Based Stress Indicators

The applied method for assessing wing morphology and asymmetry proved suitable for detecting the effects of multiple anthropogenic stressors, including the insecticide, Bti, and hydrological alterations, capturing both general and region-specific changes. Fluctuating asymmetry is often considered an indicator of environmental and genetic stress, but its predictive reliability depends on understanding evolutionary, ecological, and methodological contexts (Benítez et al. 2020). We showed, that stressors differentially affect specific facets of wing morphology and asymmetry, highlighting variation in the magnitude and nature of their impacts. In our case, simple measurements like the wing area or subtract value of the whole wings’ cells but also the wing shape or cell shape asymmetry responded to environmental changes. Other parameters, like the cell position asymmetry parameters appeared to react only in the context of the altered hydrology but correlated with wing size. We demonstrated that, depending on the environmental context, wing size can be more strongly affected than asymmetry, indicating its value as a sensitive measurement parameter. In addition, our correlation analysis (Supplementary Table S6) suggests that some of the parameters examined, such as the mean distance between cell centroids, may be positively correlated with wing size. We further included adult wet weight as an additional parameter reflecting overall body size, which also showed a positive relationship with wing size (Supplementary Table S6). However, we did not measure other morphological body size metrics such as body length. This constitutes a potential limitation, as wet weight of insects can be influenced by nutritional status, body composition, or environmental conditions (Knapp and Knappová 2013), potentially confounding its interpretation as a body size proxy. The inclusion of additional structural size measurements, such as body length, could help refine the interpretation of size-related trait responses in future studies. However, relying solely on size measurements without considering asymmetry may lead to incomplete or misleading interpretations of developmental stress, as exemplified by the insecticide treatment, where wing and cell shape asymmetry increased while wing size remained unaffected. In addition, it also highlights the necessity of combining laboratory and field approaches to disentangle direct physiological effects from community-mediated or environmentally modulated responses and to avoid misinterpretation based on isolated experimental conditions.

The duration and timing of stress are important factors determining the development of asymmetry (Beasley et al. 2013). Damselfly larvae develop their wings throughout several instars but also during the last instar, larvae go through the important process of wing sheath expansion (Okude et al. 2021), which ultimately leads to many different possible targets for stress throughout the aquatic stage. When testing multiple response variables, risk of Type II errors increases. Ideally, the interpretation of stress effects should integrate information from multiple response variables simultaneously rather than overemphasizing isolated findings. Nevertheless, especially in methodologically modern studies, isolated findings can provide valuable insights into which aspects should be examined more closely in the future. Taking the analysis one step further, this includes not only comparing simple quantities but also considering patterns such as 2-dimensional spatial changes in asymmetry of a wing. As advancements in computer-assisted trait evaluation enable the generation of a vast array of potential trait measurements, a strategic and focused approach is necessary to effectively initiate analyses.

## Conclusion

Our study demonstrates that computer-vision-assisted analysis of wing morphology and asymmetry is a suitable tool for detecting trait responses of damselflies to anthropogenic environmental changes. The insecticide increased wing shape and cell shape asymmetry, while altered hydrology affected overall wing size and cell position asymmetry. Bti exposure primarily increased cell number and tended to raise body weight. All 3 anthropogenic changes also influenced asymmetry via the subtract value of cells, although this metric was mainly driven by a few extreme values. Importantly, effects were not uniformly distributed across the wings but varied spatially and between traits. Overall wing size and asymmetry did not always respond in parallel, highlighting the need to assess multiple traits simultaneously.

As damselfly wings develop during the aquatic larval stage, their morphology can carry environmental signals into the terrestrial phase. This highlights their potential as indicators not only of aquatic ecosystem alteration but also of cross-ecosystem impacts, with possible consequences for terrestrial food webs and insect-mediated ecological functions.

Our findings contribute to a growing understanding of how agricultural pesticide use, climate-change-related hydrological shifts, and mosquito control agents may directly or indirectly affect trait expression in aquatic non-target insects. Future research should examine whether such trait alterations influence behavior, reproductive success, or species interactions and to what extent these responses are consistent across taxa and environmental gradients.

## Supplementary Material

nvaf112_Supplementary_Data

## Data Availability

Data will be made available on request.
