# Supplementary material for: Effects of chemical and hydrological stress on the wing morphology of a damselfly
Source: Environ Entomol. 2025 Nov 6;54(6):1335–47. doi: 10.1093/ee/nvaf112 (PMC12716282; doi:10.1093/ee/nvaf112)
Supplement: nvaf112_Supplementary_Data [file nvaf112_supplementary_data.zip › SI resub2 clean.docx]

Supplementary Information: Effects of chemical and hydrological stress on the wing morphology of a damselfly

Ken M. Mauser^1^*, Samiksha Paudel^1^, Olivia Sigmund^1^, Martin H. Entling^1^, Jürgen Ott², Carsten A. Brühl^1^

1 iES Landau, Institute for Environmental Sciences, University of Kaiserslautern-Landau, Landau, Germany

2 L.U.P.O. GmbH, Trippstadt, Germany


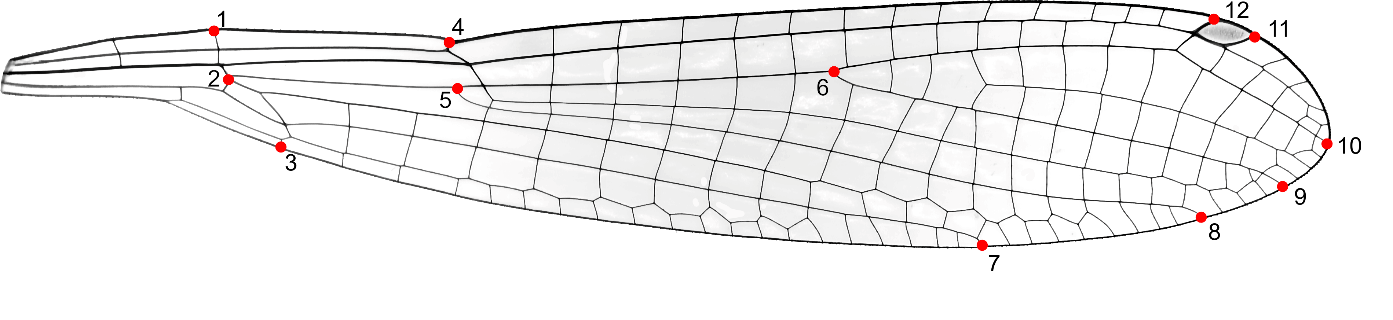


Figure S1. Position of the twelve landmarks (Lm) which were set with the software IdentiFly (version 1.5, Tofilski, 2023) on the hind antenodal cross vein (Lm 1 and 2), a branch of the anal vein (Lm 3), the nodus (Lm 4), the cubital vein (Lm 7), three branches of a medium vein (Lm 8, 9, 10) and two outer sides of the pterostigma (Lm 11, 12). Landmarks at positions 5 and 6 covered the wing interior along the 2^nd^ radius.


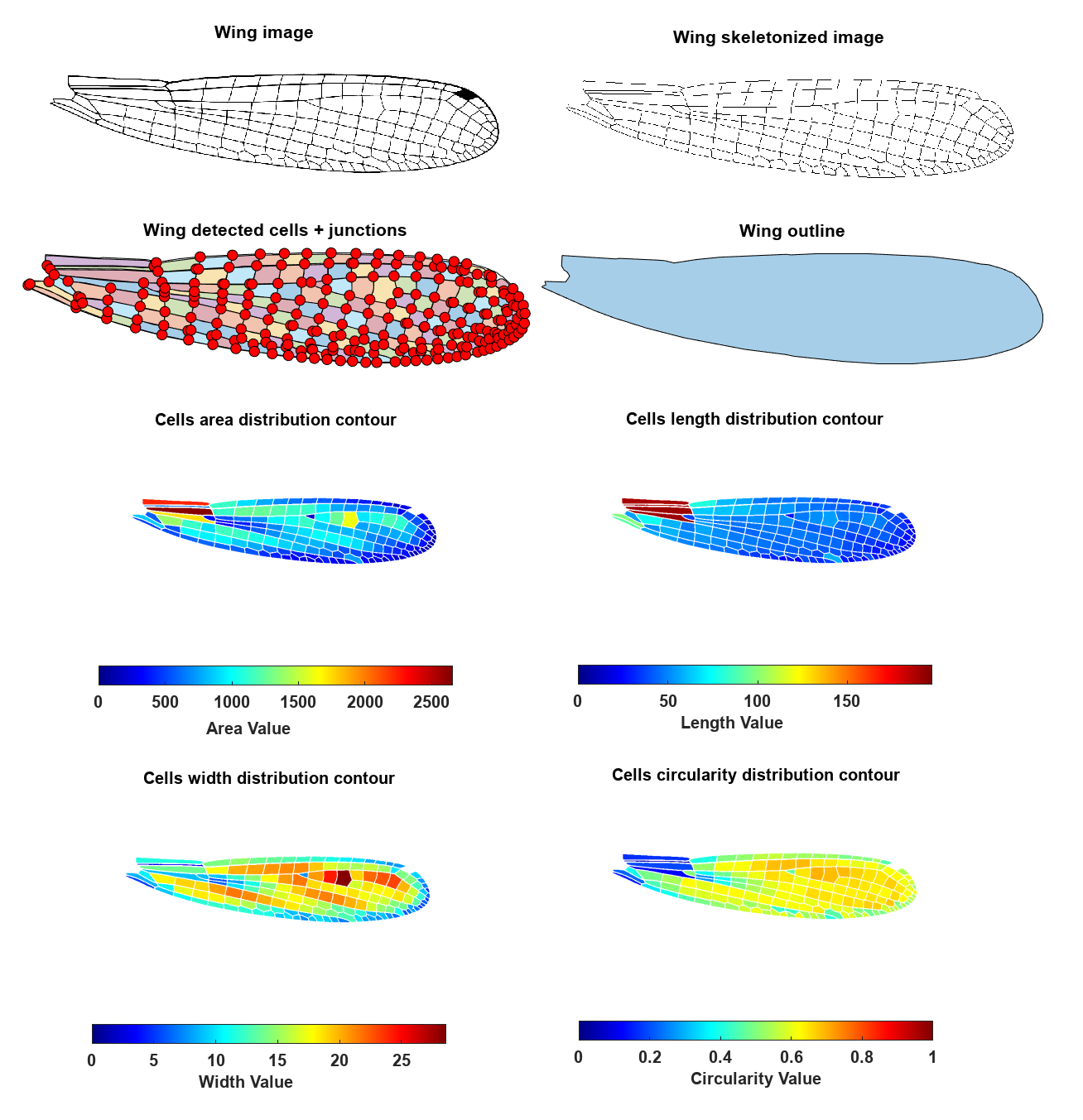


Figure S2. Illustration of the detection capabilities of WingAnalogy (Eshghi et al., 2024) computation using one right front wing as an example. Area, length and cell values in pixel. Each pixel had side length of 22.5408 μm.


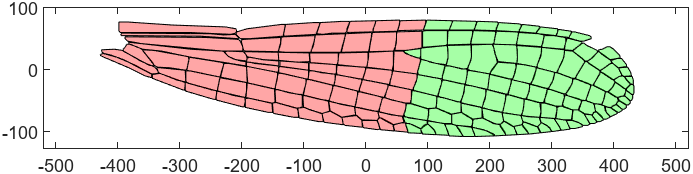

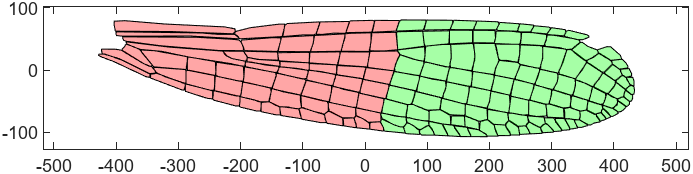


Figure S3. Separation of Set 1 (red) and Set 2 (green). Each wing was separated in two halves halves (proximal part of the wing: Set 1, distal part of the wing Set 2) at the split of the 2nd and 3rd branch of the radius, whereby cell column with the origin of the split itself was added to Set1. First cell forming between the 2nd and 3rd branch was always included in the Set 2 to maintain regularity regardless of the locally different origin of the split.


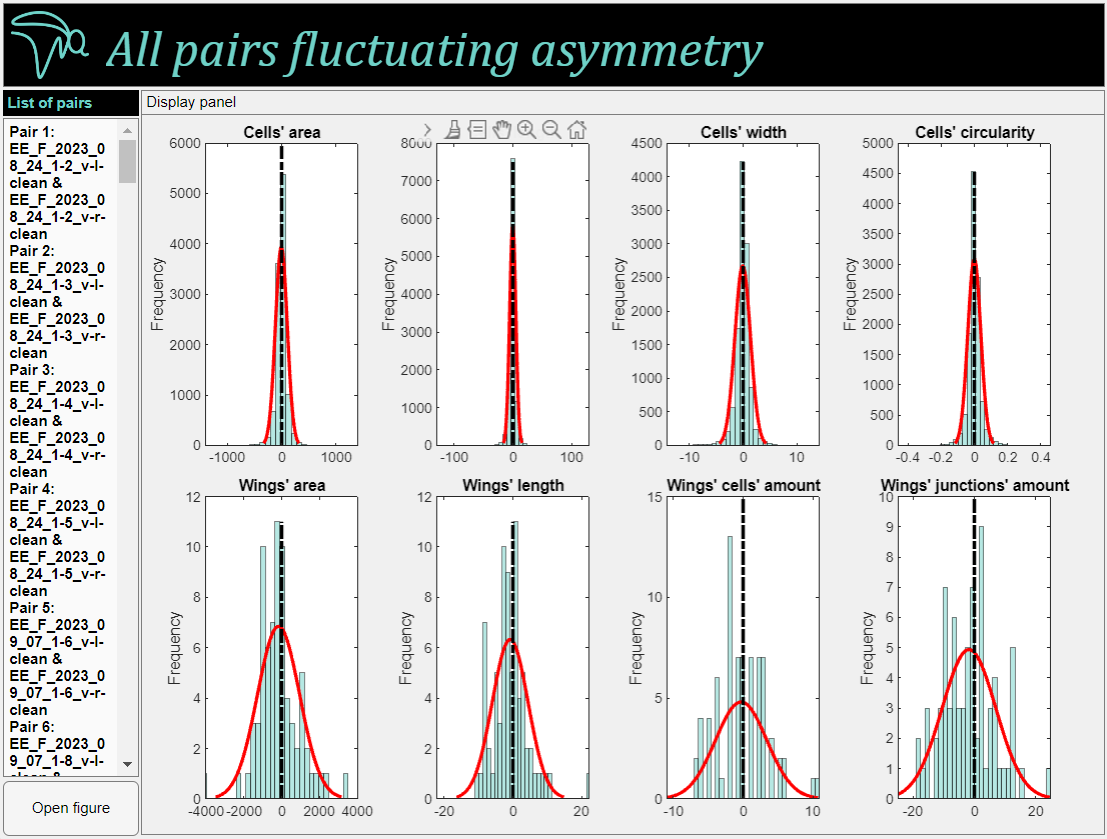


Figure S4. Front wing fluctuating asymmetry results of WingAnalogy 4.0, including cells’ area, cells’ length, cells’ width and cells’ circularity as well as wings’ area, wings’ length, number of cells and number of junctions of the mesocosm experiment. Frequence of the values on the y-axis; difference in corresponding wing or cell values [px] on the x-axis. Red line indicates the fitted normal distribution.

Table S1. Description of all investigated parameters

| Variable | Description |
| --- | --- |
| Days until emergence | Counted days from the beginning of the insecticide exposure to the date of emergence of the individual |
| Weight gain (wet) | Wet weight gain of a damselfly larva from the beginning of the insecticide exposure to the emergence |
|  |  |
| Wingload [mg/µm] | Wing load calculated as body mass per mean wing area of front or hind wings |
| Wing area [mm²] | Mean wing area between left and right wing (either front or hind wing) |
| Wing length [µm] | Mean wing length between left and right wing (either front or hind wing) measured from the base of the wing as seen in Figure S1 to the outer tip |
| Wing perimeter [µm] | Mean wing perimeter between left and right wing (either front or hind wing) |
| Wing width [µm] | Mean wing width between left and right wing (either front or hind wing) |
| Number of cells | Mean number of cells between left and right wing (either front or hind wing) |
| Number of junctions | Mean number of junctions between left and right wing (either front or hind wing) |
|  |  |
| Wings area differences | Wing area differences between left and right wing (either front or hind wing) |
| Subtract value (Junctions) | Differences in total number of junctions between left and right wing (either front or hind wing) |
| Subtract value (Cells) | Differences in total number of cells between left and right wing (either front or hind wing) |
| NRMSE(Area) | Normalized root mean squared error of the cell areas of homologous cells between the left and right wing (either front or hind wing) |
| NRMSE(Length) | Normalized root mean squared error of the cell length of homologous cells between the left and right wing (either front or hind wing) |
| NRMSE(Width) | Normalized root mean squared error of the cell width of homologous cells between the left and right wing (either front or hind wing) |
| NRMSE(Circularity) | Normalized root mean squared error of the cell circularity of homologous cells between the left and right wing (either front or hind wing). The circularity of a cell was calculated as (4 pi * area)/perimeter²) |
| Mean distance(Cells centroid) | Mean distance of cells centroid of homologous cells between the left and right wing (either front or hind wing) |
| Mean distance(Junctions) | Mean distance of junctions of homologous vein junctions between the left and right wing (either front or hind wing) |
| Mean distance(Wing outlines) | Mean distance of wing outlines between left and right wing (either front or hind wing) |
| Landmark FA-score | To calculate the FA-score, twelve landmarks were used (Figure S1). First, a procrustes fit was performed for females and males separately. FA and shape components were calculated and an individual wing pair wise FA-score was calculated as distance between FA component and mean symmetric shape. |

Table S2. Results of the climate chamber experiment. We used generalized linear models for the named response variables and the explaining variable “concentration” (0 to 0.04 mg/l) and “sex” (f, m) to investigate the impact of the insecticide concentration. For the “Days until emergence“, “Number of cells”, “Number of junctions”, “Subtract value(Cells)” and “Subtract value(Junctions)” a poisson distribution with log link was used. All other variables were fitted with a gaussian distribution with an identity link. Significancy was tested with a t-test for gaussian family models and Wald-test for poisson family models (base package core, version 4.3.2, R Core Team, 2023).

|  |  | Intercept | | | |  |  | Concentration [mg/L] | | | |  |  | Sex male | | | |  |  |
| --- | --- | --- | --- | --- | --- | --- | --- | --- | --- | --- | --- | --- | --- | --- | --- | --- | --- | --- | --- |
| **Variable** | **Wing** | **Estimate** | **Std error** | **t/z-value** | **p-value** |  |  | **Estimate** | **Std error** | **t/z-value** | **p-value** |  |  | **Estimate** | **Std error** | **t/z-value** | **p-value** |  | **family** |
| Days until emergence | - | 3.35 | 0.03 | 121.5 | **<0.001** | (***) |  | 0.92 | 1.02 | 0.90 | 0.369 |  |  | -0.04 | 0.03 | -1.28 | 0.202 |  | poisson |
| Weight gain (wet) | - | 0.69315 | 0.0299 | 23.172 | **<0.001** | (***) |  | 0.07 | 1.11 | 0.06 | 0.949 |  |  | -0.10 | 0.04 | -2.81 | **0.006** | (**) | gaussian |
|  |  |  |  |  |  |  |  |  |  |  |  |  |  |  |  |  |  |  |  |
| Wingload [mg/µm]  (weight per wing length) | front | 0.00126 | 1.5E-05 | 83.5955 | **<0.001** | (***) |  | 0.00056 | 0.00057 | 0.97302 | 0.333 |  |  | -3E-05 | 1.9E-05 | -1.80483 | 0.074 | . | gaussian |
|  | hind | 0.00136 | 1.6E-05 | 86.6984 | **<0.001** | (***) |  | 0.00105 | 0.00061 | 1.73563 | 0.086 | . |  | -5E-05 | 2E-05 | -2.37011 | **<0.001** | (***) | gaussian |
| Wingload [mg/mm²]  (weight per wing area) | front | 0.408 | 0.005 | 80.037 | **<0.001** | (***) |  | 0.222 | 0.194 | 1.145 | 0.255 |  |  | 0.015 | 0.006 | 2.390 | 0.019 |  | gaussian |
|  | hind | 0.450 | 0.005 | 84.166 | **<0.001** | (***) |  | 0.396 | 0.206 | 1.922 | 0.057 | . |  | 0.013 | 0.007 | 1.930 | 0.056 | . | gaussian |
| Wing area [mm²] | front | 56.5565 | 0.50371 | 112.28 | **<0.001** | (***) |  | 10.2887 | 19.1501 | 0.53726 | 0.592 |  |  | -7.0479 | 0.63431 | -11.1111 | **<0.001** | (***) | gaussian |
|  | hind | 50.9635 | 0.45761 | 111.37 | **<0.001** | (***) |  | 11.2128 | 17.6589 | 0.63497 | 0.527 |  |  | -6.5038 | 0.57468 | -11.3171 | **<0.001** | (***) | gaussian |
| Wing length [µm] | front | 18353.1 | 86.5123 | 212.145 | **<0.001** | (***) |  | 4435.8 | 3289.03 | 1.34866 | 0.180 |  |  | -1224.7 | 108.943 | -11.2417 | **<0.001** | (***) | gaussian |
|  | hind | 16839.1 | 79.3438 | 212.23 | **<0.001** | (***) |  | 4606.94 | 3061.85 | 1.50463 | 0.135 |  |  | -1195.7 | 99.6432 | -11.9999 | **<0.001** | (***) | gaussian |
| Wing perimeter [µm] | front | 40246.5 | 204.813 | 196.504 | **<0.001** | (***) |  | 19206.8 | 7786.59 | 2.46665 | **0.015** | (*) |  | -2655.2 | 257.917 | -10.2949 | **<0.001** | (***) | gaussian |
|  | hind | 37101.8 | 168.392 | 220.33 | **<0.001** | (***) |  | 8857.41 | 6498.18 | 1.36306 | 0.176 |  |  | -2541.2 | 211.473 | -12.0165 | **<0.001** | (***) | gaussian |
| Wing width [µm] | front | 3079.75 | 16.517 | 186.46 | **<0.001** | (***) |  | -209.575 | 627.944 | -0.33375 | 0.739 |  |  | -190.58 | 20.7995 | -9.16256 | **<0.001** | (***) | gaussian |
|  | hind | 3024.59 | 16.5094 | 183.204 | **<0.001** | (***) |  | -172.401 | 637.09 | -0.27061 | 0.787 |  |  | -184.28 | 20.7331 | -8.88815 | **<0.001** | (***) | gaussian |
| Cells amount | front | 161.093 | 1.37302 | 117.327 | **<0.001** | (***) |  | 37.0346 | 52.1997 | 0.70948 | 0.480 |  |  | -7.6506 | 1.72902 | -4.42483 | **<0.001** | (***) | poisson |
|  | hind | 148.249 | 1.21972 | 121.543 | **<0.001** | (***) |  | 43.9514 | 47.0687 | 0.93377 | 0.353 |  |  | -5.7811 | 1.53178 | -3.77408 | **<0.001** | (***) | poisson |
| Junctions amount | front | 238.933 | 2.03465 | 117.432 | **<0.001** | (***) |  | 42.4169 | 77.3534 | 0.54835 | 0.585 |  |  | -12.753 | 2.5622 | -4.97743 | **<0.001** | (***) | poisson |
|  | hind | 220.807 | 1.80106 | 122.598 | **<0.001** | (***) |  | 24.2101 | 69.5023 | 0.34834 | 0.728 |  |  | -9.9201 | 2.26185 | -4.38585 | **<0.001** | (***) | poisson |
|  |  |  |  |  |  |  |  |  |  |  |  |  |  |  |  |  |  |  |  |
| Wings area differences | front | 0.41675 | 0.0454 | 9.17952 | **<0.001** | (***) |  | -1.12602 | 1.72603 | -0.65238 | 0.516 |  |  | -0.1132 | 0.05717 | -1.98044 | 0.050 | . | gaussian |
|  | hind | 0.37487 | 0.11106 | 3.37547 | **<0.001** | (***) |  | -0.78722 | 4.28562 | -0.18369 | 0.855 |  |  | 0.15063 | 0.13947 | 1.08001 | 0.283 |  | gaussian |
| Subtract value (Junctions) | front | 1.99964 | 0.05652 | 35.3812 | **<0.001** | (***) |  | 3.95482 | 2.09347 | 1.88912 | 0.059 | . |  | -0.0927 | 0.07136 | -1.29912 | 0.194 |  | poisson |
|  | hind | 1.91364 | 0.05976 | 32.0198 | **<0.001** | (***) |  | -3.98088 | 2.49782 | -1.59374 | 0.111 |  |  | -0.1904 | 0.07931 | -2.40112 | **0.016** | (*) | poisson |
| Subtract value (Cells) | front | 1.19589 | 0.08451 | 14.1504 | **<0.001** | (***) |  | -0.70319 | 3.1391 | -0.22401 | 0.823 |  |  | 0.15652 | 0.10357 | 1.51119 | 0.131 |  | poisson |
|  | hind | 1.10533 | 0.08925 | 12.385 | **<0.001** | (***) |  | -8.87763 | 3.69471 | -2.40279 | **0.016** | (*) |  | 0.16148 | 0.11174 | 1.44517 | 0.148 |  | poisson |
| NRMSE(Area) | front | 0.32518 | 0.01139 | 28.5504 | **<0.001** | (***) |  | 0.55627 | 0.43301 | 1.28465 | 0.202 |  |  | 0.00782 | 0.01434 | 0.54527 | 0.587 |  | gaussian |
|  | hind | 0.32026 | 0.01425 | 22.479 | **<0.001** | (***) |  | -0.30561 | 0.54979 | -0.55586 | 0.580 |  |  | 0.0464 | 0.01789 | 2.59358 | **0.011** | (*) | gaussian |
| NRMSE(Area)-Set1 | front | 0.23141 | 0.01786 | 12.9593 | **<0.001** | (***) |  | 1.26108 | 0.67888 | 1.85759 | 0.066 | . |  | 0.02099 | 0.02249 | 0.93348 | 0.353 |  | gaussian |
|  | hind | 0.21407 | 0.01658 | 12.9075 | **<0.001** | (***) |  | 0.41834 | 0.64 | 0.65366 | 0.515 |  |  | 0.03112 | 0.02083 | 1.49401 | 0.138 |  | gaussian |
| NRMSE(Area)-Set2 | front | 0.43196 | 0.01424 | 30.3419 | **<0.001** | (***) |  | -0.50237 | 0.54124 | -0.92817 | 0.356 |  |  | -0.0033 | 0.01793 | -0.18146 | 0.856 |  | gaussian |
|  | hind | 0.41066 | 0.01713 | 23.9734 | **<0.001** | (***) |  | -0.0751 | 0.66103 | -0.11361 | 0.910 |  |  | 0.0462 | 0.02151 | 2.1474 | **0.034** | (*) | gaussian |
| NRMSE(Length) | front | 0.22826 | 0.00924 | 24.7162 | **<0.001** | (***) |  | 0.17347 | 0.3511 | 0.49408 | 0.622 |  |  | 0.00975 | 0.01163 | 0.83878 | 0.404 |  | gaussian |
|  | hind | 0.22606 | 0.00976 | 23.1661 | **<0.001** | (***) |  | -0.15605 | 0.37657 | -0.4144 | 0.679 |  |  | 0.02715 | 0.01225 | 2.21543 | **0.029** | (*) | gaussian |
| NRMSE(Length)-Set1 | front | 0.14164 | 0.01359 | 10.4205 | **<0.001** | (***) |  | 0.17972 | 0.51676 | 0.34779 | 0.729 |  |  | 0.01975 | 0.01712 | 1.15413 | 0.251 |  | gaussian |
|  | hind | 0.12373 | 0.01038 | 11.9186 | **<0.001** | (***) |  | 0.2223 | 0.40059 | 0.55492 | 0.580 |  |  | 0.01689 | 0.01304 | 1.29589 | 0.198 |  | gaussian |
| NRMSE(Length)-Set2 | front | 0.59594 | 0.01566 | 38.0513 | **<0.001** | (***) |  | -0.2314 | 0.59542 | -0.38864 | 0.698 |  |  | 0.02771 | 0.01972 | 1.40483 | 0.163 |  | gaussian |
|  | hind | 0.58666 | 0.01609 | 36.4698 | **<0.001** | (***) |  | 0.23624 | 0.62076 | 0.38057 | 0.704 |  |  | 0.03961 | 0.0202 | 1.96067 | 0.053 | . | gaussian |
| NRMSE(Width) | front | 0.32867 | 0.00894 | 36.7678 | **<0.001** | (***) |  | 0.028 | 0.33984 | 2.08454 | **0.040** | (*) |  | -0.0071 | 0.01126 | -0.63088 | 0.530 |  | gaussian |
|  | hind | 0.32875 | 0.01182 | 27.818 | **<0.001** | (***) |  | -0.02988 | 0.45605 | -0.06552 | 0.948 |  |  | 0.02446 | 0.01484 | 1.64837 | 0.102 |  | gaussian |
| NRMSE(Width)-Set1 | front | 0.21082 | 0.01163 | 18.1242 | **<0.001** | (***) |  | 1.14435 | 0.44221 | 2.58777 | **0.011** | (*) |  | 0.00419 | 0.01465 | 0.28618 | 0.775 |  | gaussian |
|  | hind | 0.21009 | 0.01537 | 13.6708 | **<0.001** | (***) |  | 0.1014 | 0.59303 | 0.17098 | 0.865 |  |  | 0.01972 | 0.0193 | 1.02184 | 0.309 |  | gaussian |
| NRMSE(Width)-Set2 | front | 0.38386 | 0.01277 | 30.0693 | **<0.001** | (***) |  | 0.01261 | 0.48534 | 0.02599 | 0.979 |  |  | -0.0126 | 0.01608 | -0.78319 | 0.435 |  | gaussian |
|  | hind | 0.36853 | 0.01523 | 24.1964 | **<0.001** | (***) |  | 0.01532 | 0.58775 | 0.02607 | 0.979 |  |  | 0.03385 | 0.01913 | 1.7696 | 0.080 | . | gaussian |
| NRMSE(Circularity) | front | 0.34698 | 0.00649 | 53.5023 | **<0.001** | (***) |  | 0.59396 | 0.24656 | 2.40899 | **0.018** | (*) |  | -0.0128 | 0.00817 | -1.57251 | 0.119 |  | gaussian |
|  | hind | 0.34508 | 0.00923 | 37.3869 | **<0.001** | (***) |  | -0.02543 | 0.35618 | -0.07141 | 0.943 |  |  | 0.01722 | 0.01159 | 1.48524 | 0.140 |  | gaussian |
| NRMSE(Circularity)-Set1 | front | 0.22058 | 0.00908 | 24.3013 | **<0.001** | (***) |  | 0.42321 | 0.34509 | 1.22638 | 0.223 |  |  | 0.01782 | 0.01143 | 1.55907 | 0.122 |  | gaussian |
|  | hind | 0.19161 | 0.01111 | 17.2392 | **<0.001** | (***) |  | 0.48175 | 0.42891 | 1.12321 | 0.264 |  |  | 0.02745 | 0.01396 | 1.96642 | 0.052 | . | gaussian |
| NRMSE(Circularity)-Set2 | front | 0.68761 | 0.01536 | 44.756 | **<0.001** | (***) |  | 0.71085 | 0.58409 | 1.21702 | 0.226 |  |  | -0.042 | 0.01935 | -2.16973 | **0.032** | (*) | gaussian |
|  | hind | 0.65385 | 0.0161 | 40.6087 | **<0.001** | (***) |  | -0.40171 | 0.62134 | -0.64651 | 0.519 |  |  | 0.02363 | 0.02022 | 1.16843 | 0.245 |  | gaussian |
| Mean distance(Cells centroid) | front | 124.753 | 4.26467 | 29.2526 | **<0.001** | (***) |  | -70.5701 | 162.135 | -0.43526 | 0.664 |  |  | -14.262 | 5.37043 | -2.65573 | **0.009** | (**) | gaussian |
|  | hind | 111.922 | 4.97655 | 22.4898 | **<0.001** | (***) |  | -37.4072 | 192.043 | -0.19479 | 0.846 |  |  | 4.02737 | 6.24975 | 0.6444 | 0.521 |  | gaussian |
| Mean distance(Cells centroid)-Set1 | front | 94.4009 | 4.39911 | 21.4591 | **<0.001** | (***) |  | 83.1572 | 167.246 | 0.49722 | 0.620 |  |  | -6.4188 | 5.53972 | -1.15868 | 0.249 |  | gaussian |
|  | hind | 87.9599 | 6.5168 | 13.4974 | **<0.001** | (***) |  | 126.225 | 251.481 | 0.50193 | 0.617 |  |  | -0.1859 | 8.18407 | -0.02272 | 0.982 |  | gaussian |
| Mean distance(Cells centroid)-Set2 | front | 144.045 | 5.47217 | 26.3231 | **<0.001** | (***) |  | -193.014 | 208.041 | -0.92777 | 0.356 |  |  | -18.735 | 6.89101 | -2.71872 | **0.008** | (**) | gaussian |
|  | hind | 125.557 | 5.40758 | 23.2188 | **<0.001** | (***) |  | -150.385 | 208.677 | -0.72066 | 0.473 |  |  | 8.81937 | 6.79106 | 1.29867 | 0.197 |  | gaussian |
| Mean distance(Junctions) | front | 117.46 | 3.21078 | 36.583 | **<0.001** | (***) |  | -37.5976 | 122.068 | -0.30801 | 0.759 |  |  | -12.077 | 4.04329 | -2.98699 | **0.004** | (**) | gaussian |
|  | hind | 107.598 | 3.89701 | 27.6103 | **<0.001** | (***) |  | -10.1865 | 150.384 | -0.06774 | 0.946 |  |  | 2.00202 | 4.89402 | 0.40907 | 0.683 |  | gaussian |
| Mean distance(Wing outlines) | front | 26.011 | 1.43679 | 18.1035 | **<0.001** | (***) |  | 86.317 | 54.6241 | 1.5802 | 0.117 |  |  | -2.0234 | 1.80933 | -1.11833 | 0.266 |  | gaussian |
|  | hind | 27.4822 | 3.83979 | 7.15722 | **<0.001** | (***) |  | -56.7505 | 148.176 | -0.38299 | 0.703 |  |  | 5.12778 | 4.82216 | 1.06338 | 0.290 |  | gaussian |
| Landmark FA-score | front | 0.01433 | 0.00087 | 16.4154 | **<0.001** | (***) |  | 0.08336 | 0.03349 | 2.48907 | **0.014** | (*) |  | -0.0013 | 0.0011 | -1.1472 | 0.254 |  | gaussian |
|  | hind | 0.01512 | 0.00099 | 15.2108 | **<0.001** | (***) |  | 0.00948 | 0.03767 | 0.25156 | 0.802 |  |  | 0.00126 | 0.00123 | 1.02883 | 0.306 |  | gaussian |

Table S3. Results of the floodplain mesocosm experiment. The stressors treatment (non-bti/bti), hydrology (control/altered hydrology), their interaction and sex (f/m) were used inside generalized linear models as explaining variables. A Likelihood-Ratio Chi² tests (car package, version 3.1.2, Fox and Weisberg, 2019) was applied to assess the overall effect of categorical predictors and interactions in the mesocosm models.

|  |  | Intercept | |  | Treatment (non-bti/bti) | | | |  | Hydrological regime (control/scenario) | | | |  | Interaction(treatment Bti/hydrological regime: altered) | | | |  | Sex (f/m) | | | |  |
| --- | --- | --- | --- | --- | --- | --- | --- | --- | --- | --- | --- | --- | --- | --- | --- | --- | --- | --- | --- | --- | --- | --- | --- | --- |
| **Variable** | **Wing** | **Estimate** | **Std Error** |  | **Estimate** | **Std Error** | **Chisq** | **p-value** |  | **Estimate** | **Std Error** | **Chisq** | **p-value** |  | **Estimate** | **Std Error** | **Chisq** | **p-value** |  | **Estimate** | **Std Error** | **Chisq** | **p-value** |  |
| **Body weight wet [mg]** | **-** | **28.26** | **1.33** |  | **1.17** | **1.74** | **2.98** | **0.084** | **.** | **3.36** | **1.91** | **12.04** | **<0.001** | **(***)** | **2.90** | **2.75** | **52.53** | **<0.001** | **(***)** | **-9.97** | **1.38** | **52.53** | **<0.001** | **(***)** |
| Wingload [mg/µm]  (weight per wing length) | front | 0.002 | 0.0001 |  | 0.00002 | 0.0001 | 1.21 | 0.271 |  | 0.0004 | 0.0001 | 26.24 | **<0.001** | (***) | 0.0002 | 0.0002 | 1.47 | 0.225 |  | -0.0006 | 0.0001 | 45.92 | **<0.001** | (***) |
|  | hind | 0.002 | 0.0001 |  | 0.00002 | 0.0001 | 0.76 | 0.384 |  | 0.0004 | 0.0002 | 24.80 | **<0.001** | (***) | 0.0002 | 0.0002 | 1.01 | 0.316 |  | -0.0007 | 0.0001 | 44.29 | **<0.001** | (***) |
| Wingload [mg/mm²]  (weight per wing area) | front | 0.54 | 0.03 |  | 0.01 | 0.03 | 1.13 | 0.288 |  | 0.10 | 0.04 | 22.53 | **<0.001** | (***) | 0.05 | 0.05 | 1.06 | 0.304 |  | -0.15 | 0.03 | 34.38 | **<0.001** | (***) |
|  | hind | 0.59 | 0.03 |  | 0.01 | 0.03 | 0.90 | 0.344 |  | 0.11 | 0.04 | 20.20 | **<0.001** | (***) | 0.06 | 0.06 | 0.87 | 0.350 |  | -0.16 | 0.03 | 30.86 | **<0.001** | (***) |
| Wing area [mm²] | front | 57.69 | 1.20 |  | 0.66 | 1.38 | 0.52 | 0.473 |  | 5.33 | 1.77 | 20.42 | **<0.001** | (***) | 0.43 | 2.41 | 0.03 | 0.858 |  | -10.88 | 1.18 | 85.01 | **<0.001** | (***) |
|  | hind | 52.58 | 1.01 |  | 0.68 | 1.21 | 0.12 | 0.727 |  | 5.91 | 1.59 | 23.06 | **<0.001** | (***) | -1.10 | 2.20 | 0.25 | 0.619 |  | -10.57 | 1.04 | 103.28 | **<0.001** | (***) |
| Wing length [µm] | front | 18451.63 | 193.94 |  | 240.94 | 222.34 | 0.75 | 0.385 |  | 998.70 | 284.71 | 19.11 | **<0.001** | (***) | -251.65 | 387.86 | 0.42 | 0.517 |  | -1913.01 | 189.98 | 101.40 | **<0.001** | (***) |
|  | hind | 16958.66 | 175.94 |  | 259.02 | 210.68 | 0.48 | 0.487 |  | 1079.30 | 277.68 | 19.08 | **<0.001** | (***) | -450.00 | 383.95 | 1.37 | 0.241 |  | -1886.81 | 181.28 | 108.33 | **<0.001** | (***) |
| Wing perimeter [µm] | front | 40381.50 | 413.08 |  | 456.81 | 473.59 | 0.68 | 0.410 |  | 2146.03 | 606.42 | 20.83 | **<0.001** | (***) | -417.10 | 826.15 | 0.25 | 0.614 |  | -4060.00 | 404.65 | 100.67 | **<0.001** | (***) |
|  | hind | 37352.24 | 376.49 |  | 494.14 | 450.82 | 0.35 | 0.556 |  | 2356.48 | 594.20 | 20.83 | **<0.001** | (***) | -896.96 | 821.59 | 1.19 | 0.275 |  | -4077.73 | 387.91 | 110.50 | **<0.001** | (***) |
| Wing width [µm] | front | 3111.91 | 35.84 |  | 2.84 | 41.09 | 0.39 | 0.530 |  | 128.07 | 52.62 | 18.32 | **<0.001** | (***) | 54.39 | 71.68 | 0.58 | 0.448 |  | -282.84 | 35.11 | 64.89 | **<0.001** | (***) |
|  | hind | 3085.92 | 30.89 |  | 3.50 | 36.99 | 0.03 | 0.866 |  | 158.82 | 48.76 | 22.59 | **<0.001** | (***) | 5.50 | 67.41 | 0.01 | 0.935 |  | -299.68 | 31.83 | 88.65 | **<0.001** | (***) |
| Cells amount | front | 154.06 | 2.36 |  | 3.88 | 2.71 | 4.75 | **0.029** | (*) | -0.56 | 3.47 | 0.14 | 0.711 |  | 2.77 | 4.73 | 0.34 | 0.558 |  | -6.99 | 2.32 | 9.11 | **0.003** | (**) |
|  | hind | 144.94 | 2.13 |  | 2.87 | 2.55 | 2.15 | 0.142 |  | -0.28 | 3.36 | 0.00 | 0.956 |  | 0.78 | 4.64 | 0.03 | 0.866 |  | -9.03 | 2.19 | 16.94 | **<0.001** | (***) |
| Junctions amount | front | 226.83 | 3.28 |  | 4.44 | 3.76 | 2.88 | 0.090 | . | 4.29 | 4.81 | 2.67 | 0.102 |  | 2.25 | 6.56 | 0.12 | 0.731 |  | -9.97 | 3.21 | 9.64 | **0.002** | (**) |
|  | hind | 214.25 | 3.03 |  | 3.59 | 3.62 | 1.05 | 0.306 |  | 5.91 | 4.78 | 2.29 | 0.130 |  | -1.68 | 6.60 | 0.06 | 0.800 |  | -13.81 | 3.12 | 19.61 | **<0.001** | (***) |
|  |  |  |  |  |  |  |  |  |  |  |  |  |  |  |  |  |  |  |  |  |  |  |  |  |
| Wings area differences | front | 0.30 | 0.10 |  | 0.02 | 0.11 | 0.12 | 0.728 |  | 0.14 | 0.14 | 2.75 | 0.097 | . | 0.04 | 0.19 | 0.03 | 0.855 |  | 0.11 | 0.09 | 1.33 | 0.248 |  |
|  | hind | 0.42 | 0.14 |  | 0.23 | 0.17 | 0.58 | 0.446 |  | 0.14 | 0.23 | 0.15 | 0.702 |  | -0.39 | 0.31 | 1.55 | 0.213 |  | -0.13 | 0.15 | 0.83 | 0.362 |  |
| Subtract value (Junctions) | front | 1.59 | 0.11 |  | 0.18 | 0.12 | 1.32 | 0.250 |  | 0.62 | 0.13 | 31.25 | **<0.001** | (***) | -0.18 | 0.18 | 1.03 | 0.310 |  | 0.16 | 0.09 | 2.85 | 0.091 | . |
|  | hind | 1.79 | 0.10 |  | -0.01 | 0.12 | 3.50 | 0.061 | . | 0.47 | 0.13 | 6.36 | **0.012** | (*) | -0.46 | 0.20 | 5.36 | **0.021** | (*) | -0.14 | 0.10 | 2.14 | 0.144 |  |
| Subtract value (Cells) | front | 0.58 | 0.17 |  | -0.04 | 0.18 | 0.08 | 0.783 |  | 0.38 | 0.21 | 10.89 | **0.001** | (**) | 0.19 | 0.28 | 0.47 | 0.491 |  | 0.57 | 0.15 | 14.78 | **<0.001** | (***) |
|  | hind | 1.07 | 0.13 |  | 0.29 | 0.15 | 6.49 | **0.011** | **(*)** | -0.19 | 0.23 | 0.68 | 0.410 |  | 0.12 | 0.29 | 0.17 | 0.677 |  | 0.08 | 0.13 | 0.37 | 0.544 |  |
| NRMSE(Area) | front | 0.31 | 0.02 |  | -0.01 | 0.02 | 1.49 | 0.223 |  | 0.04 | 0.03 | 0.58 | 0.446 |  | -0.04 | 0.04 | 1.05 | 0.305 |  | 0.00 | 0.02 | 0.06 | 0.806 |  |
|  | hind | 0.30 | 0.02 |  | 0.04 | 0.03 | 0.77 | 0.381 |  | 0.04 | 0.04 | 0.28 | 0.596 |  | -0.06 | 0.05 | 1.19 | 0.276 |  | 0.03 | 0.02 | 1.83 | 0.176 |  |
| NRMSE(Area)-Set1 | front | 0.23 | 0.03 |  | -0.01 | 0.03 | 0.00 | 0.970 |  | -0.01 | 0.04 | 0.00 | 0.956 |  | 0.02 | 0.05 | 0.14 | 0.705 |  | 0.02 | 0.02 | 0.37 | 0.542 |  |
|  | hind | 0.21 | 0.03 |  | 0.06 | 0.04 | 2.45 | 0.118 |  | 0.04 | 0.05 | 0.16 | 0.692 |  | -0.05 | 0.07 | 0.49 | 0.482 |  | 0.03 | 0.03 | 0.79 | 0.374 |  |
| NRMSE(Area)-Set2 | front | 0.39 | 0.03 |  | 0.00 | 0.03 | 0.94 | 0.333 |  | 0.07 | 0.04 | 1.17 | 0.280 |  | -0.07 | 0.05 | 1.62 | 0.203 |  | 0.00 | 0.03 | 0.01 | 0.931 |  |
|  | hind | 0.40 | 0.03 |  | 0.03 | 0.03 | 0.08 | 0.772 |  | 0.07 | 0.04 | 0.85 | 0.355 |  | -0.08 | 0.06 | 1.95 | 0.163 |  | 0.03 | 0.03 | 1.26 | 0.262 |  |
| NRMSE(Length) | front | 0.22 | 0.01 |  | -0.01 | 0.01 | 0.80 | 0.370 |  | 0.02 | 0.02 | 2.91 | 0.088 | . | 0.00 | 0.02 | 0.00 | 0.978 |  | 0.01 | 0.01 | 1.48 | 0.224 |  |
|  | hind | 0.21 | 0.01 |  | 0.01 | 0.01 | 0.01 | 0.933 |  | 0.03 | 0.02 | 2.78 | 0.096 | . | -0.02 | 0.02 | 0.60 | 0.440 |  | 0.01 | 0.01 | 1.10 | 0.294 |  |
| NRMSE(Length)-Set1 | front | 0.13 | 0.02 |  | -0.01 | 0.02 | 0.13 | 0.721 |  | 0.00 | 0.02 | 2.71 | 0.100 | . | 0.05 | 0.03 | 2.04 | 0.153 |  | 0.02 | 0.02 | 1.74 | 0.187 |  |
|  | hind | 0.12 | 0.02 |  | 0.02 | 0.02 | 0.81 | 0.369 |  | 0.02 | 0.03 | 0.64 | 0.423 |  | -0.01 | 0.04 | 0.16 | 0.693 |  | 0.01 | 0.02 | 0.15 | 0.699 |  |
| NRMSE(Length)-Set2 | front | 0.59 | 0.03 |  | -0.01 | 0.03 | 2.12 | 0.145 |  | 0.06 | 0.04 | 1.27 | 0.259 |  | -0.07 | 0.05 | 1.66 | 0.198 |  | 0.01 | 0.03 | 0.12 | 0.734 |  |
|  | hind | 0.58 | 0.02 |  | 0.00 | 0.03 | 0.20 | 0.653 |  | 0.07 | 0.04 | 2.12 | 0.146 |  | -0.05 | 0.05 | 0.83 | 0.361 |  | 0.02 | 0.03 | 0.39 | 0.530 |  |
| NRMSE(Width) | front | 0.31 | 0.02 |  | -0.02 | 0.02 | 2.82 | 0.093 | . | 0.03 | 0.02 | 1.04 | 0.308 |  | -0.02 | 0.03 | 0.39 | 0.532 |  | 0.00 | 0.02 | 0.09 | 0.763 |  |
|  | hind | 0.32 | 0.02 |  | 0.02 | 0.02 | 0.17 | 0.684 |  | 0.03 | 0.03 | 0.29 | 0.593 |  | -0.04 | 0.04 | 0.94 | 0.332 |  | 0.01 | 0.02 | 0.17 | 0.678 |  |
| NRMSE(Width)-Set1 | front | 0.22 | 0.02 |  | -0.03 | 0.03 | 0.70 | 0.404 |  | -0.03 | 0.03 | 0.19 | 0.663 |  | 0.03 | 0.05 | 0.46 | 0.499 |  | 0.02 | 0.02 | 0.69 | 0.405 |  |
|  | hind | 0.19 | 0.03 |  | 0.06 | 0.03 | 3.62 | 0.057 | . | 0.01 | 0.04 | 0.28 | 0.594 |  | -0.04 | 0.06 | 0.48 | 0.489 |  | 0.01 | 0.03 | 0.18 | 0.674 |  |
| NRMSE(Width)-Set2 | front | 0.34 | 0.02 |  | 0.00 | 0.02 | 0.42 | 0.519 |  | 0.05 | 0.03 | 1.21 | 0.271 |  | -0.04 | 0.04 | 1.14 | 0.286 |  | 0.01 | 0.02 | 0.06 | 0.802 |  |
|  | hind | 0.36 | 0.02 |  | 0.02 | 0.03 | 0.01 | 0.929 |  | 0.06 | 0.04 | 1.25 | 0.263 |  | -0.06 | 0.05 | 1.37 | 0.243 |  | 0.02 | 0.02 | 0.72 | 0.398 |  |
| NRMSE(Circularity) | front | 0.33 | 0.01 |  | 0.00 | 0.01 | 0.03 | 0.864 |  | 0.01 | 0.02 | 1.72 | 0.190 |  | 0.01 | 0.02 | 0.04 | 0.833 |  | 0.00 | 0.01 | 0.14 | 0.705 |  |
|  | hind | 0.32 | 0.01 |  | 0.01 | 0.02 | 0.53 | 0.467 |  | 0.02 | 0.02 | 1.43 | 0.232 |  | -0.01 | 0.03 | 0.12 | 0.730 |  | 0.02 | 0.01 | 1.67 | 0.196 |  |
| NRMSE(Circularity)-Set1 | front | 0.21 | 0.02 |  | 0.00 | 0.02 | 0.02 | 0.891 |  | 0.00 | 0.03 | 0.30 | 0.585 |  | 0.01 | 0.04 | 0.10 | 0.756 |  | 0.01 | 0.02 | 0.41 | 0.523 |  |
|  | hind | 0.18 | 0.01 |  | 0.02 | 0.02 | 1.99 | 0.158 |  | 0.01 | 0.02 | 0.21 | 0.645 |  | -0.01 | 0.03 | 0.11 | 0.744 |  | 0.02 | 0.01 | 2.53 | 0.112 |  |
| NRMSE(Circularity)-Set2 | front | 0.71 | 0.03 |  | 0.03 | 0.03 | 0.82 | 0.367 |  | 0.03 | 0.04 | 0.84 | 0.360 |  | -0.01 | 0.05 | 0.03 | 0.872 |  | -0.04 | 0.03 | 1.75 | 0.185 |  |
|  | hind | 0.66 | 0.03 |  | 0.01 | 0.04 | 0.00 | 0.953 |  | 0.05 | 0.05 | 0.87 | 0.352 |  | -0.04 | 0.06 | 0.34 | 0.561 |  | 0.04 | 0.03 | 2.07 | 0.150 |  |
| Mean distance(Cells centroid) | front | 126.49 | 7.34 |  | -4.13 | 8.42 | 0.68 | 0.409 |  | 1.28 | 10.78 | 0.02 | 0.882 |  | -4.54 | 14.68 | 0.10 | 0.757 |  | -5.31 | 7.19 | 0.55 | 0.460 |  |
|  | hind | 110.80 | 6.31 |  | 5.82 | 7.55 | 0.11 | 0.738 |  | 21.28 | 9.96 | 4.64 | **0.031** | (*) | -12.18 | 13.77 | 0.78 | 0.377 |  | -7.48 | 6.50 | 1.33 | 0.250 |  |
| Mean distance(Cells centroid)-Set1 | front | 97.80 | 8.32 |  | -3.75 | 9.53 | 0.20 | 0.654 |  | 1.34 | 12.21 | 0.04 | 0.833 | . | 0.85 | 16.63 | 0.00 | 0.959 |  | 5.53 | 8.15 | 0.46 | 0.497 |  |
|  | hind | 90.24 | 7.56 |  | 7.33 | 9.05 | 0.23 | 0.628 |  | 13.92 | 11.93 | 0.85 | 0.358 |  | -12.09 | 16.49 | 0.54 | 0.464 |  | -1.94 | 7.79 | 0.06 | 0.803 |  |
| Mean distance(Cells centroid)-Set2 | front | 138.24 | 8.76 |  | 1.95 | 10.04 | 0.09 | 0.767 |  | 6.20 | 12.85 | 0.01 | 0.936 |  | -13.11 | 17.51 | 0.56 | 0.454 |  | -15.17 | 8.58 | 3.13 | 0.077 | . |
|  | hind | 122.55 | 7.76 |  | 6.59 | 9.29 | 0.16 | 0.689 |  | 23.89 | 12.25 | 4.42 | **0.036** | (*) | -11.45 | 16.93 | 0.46 | 0.499 |  | -10.38 | 7.99 | 1.69 | 0.194 |  |
| Mean distance(Junctions) | front | 118.37 | 5.84 |  | -2.53 | 6.70 | 0.49 | 0.485 |  | 2.93 | 8.57 | 0.02 | 0.878 |  | -3.82 | 11.68 | 0.11 | 0.744 |  | -4.88 | 5.72 | 0.73 | 0.394 |  |
|  | hind | 105.64 | 4.92 |  | 6.35 | 5.90 | 0.39 | 0.535 |  | 16.37 | 7.77 | 3.94 | **0.047** | (*) | -10.84 | 10.74 | 1.02 | 0.313 |  | -5.61 | 5.07 | 1.22 | 0.269 |  |
| Mean distance(Wing outlines) | front | 28.54 | 2.45 |  | -3.89 | 2.81 | 2.78 | 0.095 | . | 0.00 | 3.60 | 0.00 | 0.959 |  | 0.25 | 4.90 | 0.00 | 0.960 |  | 0.33 | 2.40 | 0.02 | 0.892 |  |
|  | hind | 25.91 | 3.82 |  | 6.04 | 4.57 | 0.82 | 0.366 |  | 0.88 | 6.02 | 0.71 | 0.398 |  | -8.54 | 8.33 | 1.05 | 0.305 |  | -1.13 | 3.93 | 0.08 | 0.775 |  |
| Landmark FA-score | front | 0.014 | 0.002 |  | 0.000 | 0.002 | 0.18 | 0.671 |  | 0.001 | 0.003 | 0.56 | 0.456 |  | 0.00 | 0.00 | 0.11 | 0.739 |  | 0.00 | 0.00 | 0.75 | 0.386 |  |
|  | hind | 0.016 | 0.001 |  | 0.000 | 0.002 | 0.20 | 0.656 |  | -0.001 | 0.002 | 0.04 | 0.837 |  | 0.00 | 0.00 | 0.47 | 0.494 |  | 0.00 | 0.00 | 1.45 | 0.229 |  |


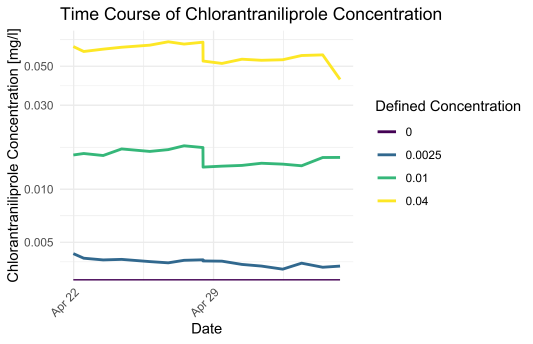


Figure S5. Time course of the measured chlorantraniliprole concentration over the first few days of the exposition. Water was added to compensate for evaporation at half-time of the experiment.


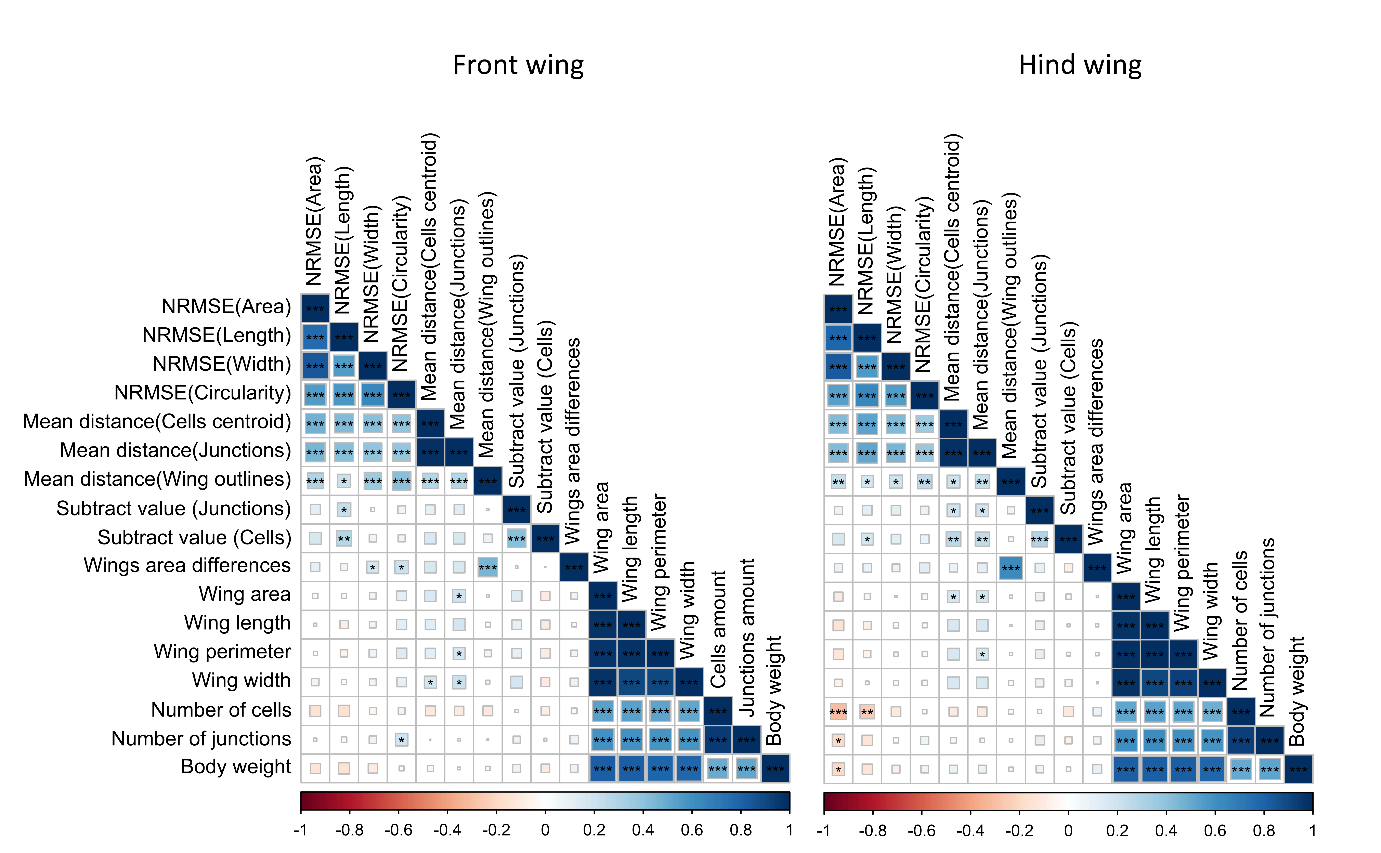


Figure S6. Spearman correlation plot for the variables NRMSE of cell area, length, width and circularity, mean distance of cells centroid, junctions and wing outlines, the subtract values of cells and junctions, wing area differences wing area, length, perimeter and width, cells and junctions amount and body weight. Reddish colour indicates a negative, blueish a positive Spearman correlation. Squares with a star indicate a significant spearman correlation depending on the significance level (p < 0.05 = *, p < 0.01 = **, p < 0.001 = ***).


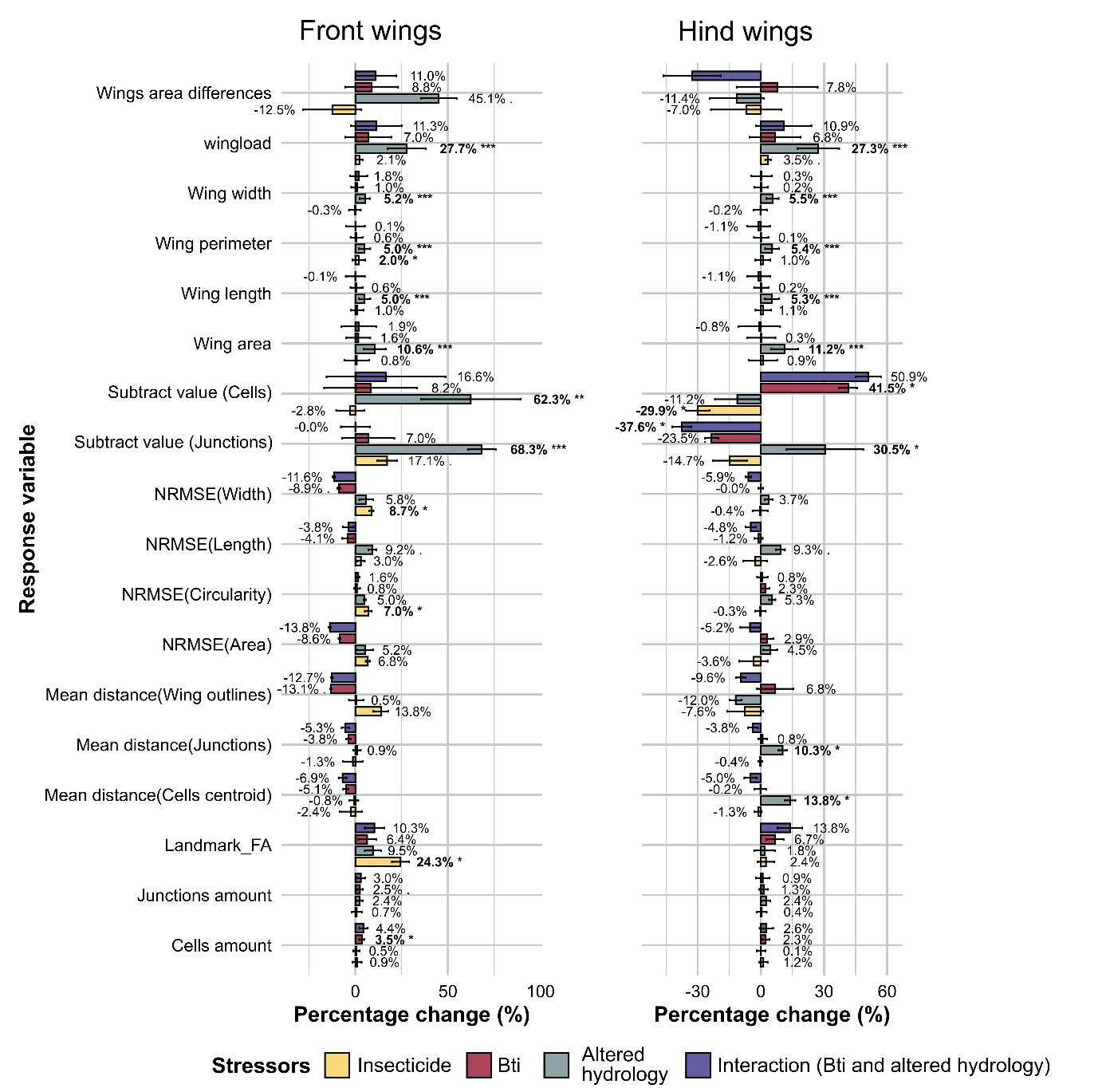


Figure S7. The three stressors insecticide (yellow), Bti (red), altered hydrology (grey) and interaction of Bti and altered hydrology (purple) and their significant effect sizes are shown as predicted percentage changes relative to the respective control group. Respective percentage changes are averaged over the levels of the other factors, thus also representing both sexes. Error bars indicate standard errors based on variation in model predictions. Interaction values represent the effect of Bti compared to the control group, calculated under altered hydrology. Values refer to different response variables of the wing size (load, width, perimeter, length, area and number of cells) and wing asymmetry (Cell shape asymmetry: NRMSE(Width), NRMSE(Circularity); cell position asymmetry: Mean distance of cells centroids and junctions, wing shape asymmetry: FA-score; count asymmetry: Subtract values). The predicted effect of the insecticide applies to a concentration of 0.04 mg/L relative to the control (0 mg/L). To test for effects of the insecticide, a generalized linear model was constructed for each response variable, with insecticide concentration and sex as explanatory variables. To test for effects of Bti and the altered hydrology, a Likelihood-Ratio Chi² test was conducted for generalized linear models of each response variable, with Bti treatment, altered hydrology, their interaction and sex as explanatory variables.
